# Design, synthesis, and biological activity of novel 1,2,4-oxadiazole derivatives

**DOI:** 10.1186/s13065-020-00722-1

**Published:** 2020-11-22

**Authors:** Lingzhi Zhu, Huanan Zeng, Dan Liu, Yun Fu, Qiong Wu, Baoan Song, Xiuhai Gan

**Affiliations:** State Key Laboratory Breeding Base of Green Pesticide and Agricultural Bioengineering, Key Laboratory of Green Pesticide and Agricultural Bioengineering, Ministry of Education, Guizhou University, Guiyang, 550025 China

**Keywords:** Synthesis, 1,2,4-Oxadiazole derivatives, Trifluoromethyl pyridine, Antibacterial activity, Nematocidal activity

## Abstract

**Background:**

Plant diseases seriously threaten food security, it is urgent to discover efficient and low-risk chemical pesticides. 1,2,4-Oxadiazole derivatives exhibit broad spectrum of agricultural biological activities. For discovering novel molecules with excellent agricultural activities, novel 1,2,4-oxadiazole derivatives were synthesized and evaluated for their agricultural activities.

**Result:**

Bioassays results showed that the title compounds exhibited moderate nematocidal activity against *Meloidogyne incognita* and anti-fungal activity to *Rhizoctonia solani*. It’s worth noting that compounds **5m**, **5r**, **5u**, **5v**, **5x** and **5y** showed strong antibacterial effects on *Xanthomonas oryzae* pv. *oryzae* (*Xoo*), with EC_50_ values of 36.25, 24.14, 28.82, 19.44, 25.37 and 28.52 μg/mL, respectively, superior to bismerthiazol (BMT, EC_50_ = 77.46 μg/mL) and thiodiazole copper (TDC, EC_50_ = 99.31 μg/mL). Compounds **5p**, **5u** and **5v** exhibited excellent antibacterial ability against *Xanthomonas oryzae* pv. *oryzicola* (*Xoc*), with EC_50_ values of 31.40, 19.04 and 21.78 μg/mL, respectively, better than that of BMT (EC_50_ = 68.50 μg/mL) and TDC (EC_50_ = 91.05 μg/mL). In addition, compound **5v** exerted moderate antibacterial effects on rice bacterial leaf blight.

**Conclusions:**

Twenty-six novel 1,2,4-oxadiazole derivatives were obtained and their biological activities were evaluated. Compound **5u** and **5v** exhibited excellent antibacterial activity *Xoo* and *Xoc.* These results indicated that 1,2,4-oxadiazole derivatives containing a trifluoromethyl pyridine moiety could be as potential alternative templates for discovering novel antibacterial agents.
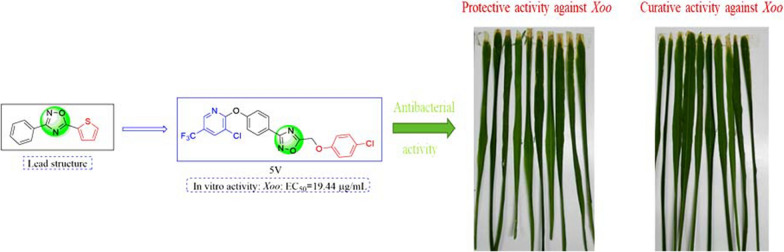

## Introduction

Crop plants are constantly challenged by a wide variety of pathogens which threaten their growth and survival, such as bacteria, fungus, and plant-parasitic nematodes. As two rice bacterial diseases, rice bacterial leaf blight and rice bacterial leaf streaks caused by *Xanthomonas oryzae* pv. *oryzae* (*Xoo*) and *Xanthomonas oryzae* pv. *oryzicola* (*Xoc*), respectively, serious impact on every stage of plant growth and development. These diseases may result in a loss of up to 80% of the crop and cause severe economic damage [1–4]. Meanwhile, fungal diseases, for example, rice sheath wilt caused by *Rhizoctonia solani*, still pose a huge threat to global agriculture [5]. In addition, over 3000 plant species are affected by nematodes worldwide, including ornamental flowers, fruit trees, cereals and vegetables [6–8]. The diseases caused by nematodes infecting plants are a serious threat to crop security, causing over $157 billion in economic losses to farmers worldwide [9, 10]. Root-knot nematode is a plant parasitic nematode that affects plant growth by essentially damaging the plant roots [11, 12]. There is an urgent need to devise a method for the effective manual control of these plant diseases, as plants cannot quickly and effectively resist them [13]. Presently, pesticides are often used for agricultural control due to their rapid response to plant diseases [14, 15], however, the long-term abuse of pesticides has led to the emergence of resistance in pathogenic organisms and may pose a risk to human health [16–18]. Therefore, developing novel, highly-efficient, and environmentally benign agents against plant diseases remains a daunting task in pesticide sciences.

**Fig. 1:**
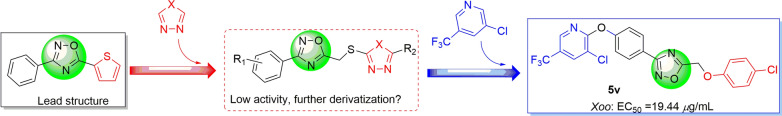
Design strategy of the target compounds

Heterocyclic structures are widely used in molecular design, and many commodity medicines have been developed [19], such as tioxazafen (Fig. 1), bismerthiazol and fluopyram. As an important five-membered heterocyclic scaffold, 1,2,4-oxadiazoles, with good potent biological properties [20, 21] have been extensively used in pesticide and medicine [22–24] molecule design. Moreover, the 1,2,4-oxadiazole heterocycle is a bioisostere of amide but shows better hydrolytic and metabolic stability [22], it is still used as an important pharmacophore to create novel drug molecules. Meanwhile, 1,3,4-thiadiazol and 1,3,4-oxadiazole have been reported to have good biological activities and were used to design drug molecules in pesticide. In our previous work, we designed and synthesized a series of novel 1,3,4-thiadiazol and 1,3,4-oxadiazole derivatives with effective control of bacterial [25, 26], fungal [27–29] and plant-parasitic nematodes [30] diseases. In addition, trifluoromethyl pyridine is an important heterocyclic structure containing fluorine, and also a common group in the current commercial pesticides. Fluopyram, containing a trifluoromethyl pyridine moiety, is not only used for control of fungal diseases, but also is used for control plant-parasitic nematodes disease [31, 32].

From the above standpoints, the compounds containing an 1,2,4-oxadiazole heterocycle, 1,3,4-thiadiazol (1,3,4-oxadiazole), or trifluoromethyl pyridine moiety exhibit broad-spectrum agricultural biological activities, which can be used as pharmacophore to design the novel pesticide. Encouraged by these promising results, and in order to obtain compounds with higher biological activity, we employed the structure-based bioisostere strategy, an excellent tool for lead were introduced into 1,2,4-optimisation, 1,3,4-thiadiazol (1,3,4-oxadiazole) and trifluoromethyl pyridine pharmacophores oxadiazole skeleton to design and synthesize a series of novel 1,2,4-oxadiazole derivatives. Meanwhile their agricultural biological activities, including nematocidal, anti-fungal, and antibacterial activity were roundly evaluated. We aimed to discovery novel structure diversity molecules with broad-spectrum activity for development of new pesticides.

## Methods

### Chemistry

All reagents and chemical materials of the analytically pure were purchased from chemical commercial companies. The reactions were monitored by thin-layer chromatography analysis and the ZF_7_ ultraviolet analyzer (Yuhua Instrument Co., Ltd. Gong Yi, China). ^1^H and ^13^C NMR spectra were obtained on the JEOL-ECX-500 spectrometer (JEOL, Tokyo, Japan) or 400 MHz spectrometer (JEOL, Tokyo, Japan). The melting points of the compounds were measurement by the X-4B melting point instrument of readings were uncorrected (Yidian Physical Optical Instrument Co., Ltd. Shanghai, China). High-resolution mass spectra (ESI TOF (+)) were obtained on the LTQ Orbitrap XL (Thermo Scientific, MO, USA).

#### General synthesis procedure for compounds **5a–5i**

**Scheme 1:**
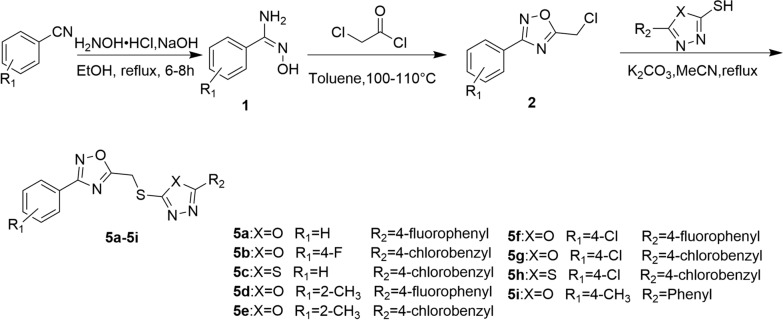
Synthesis of target compounds **5a–5i**

The procedure for synthesizing the target compounds **5a–5i** was described in Scheme 1. A mixture of substituted benzonitrile (5.0 mmol), NaOH (3.0 mmol) and hydroxylamine hydrochloride (7.5 mmol) in ethanol/water (30 mL, *V:V* = 5:1) was refluxed for 4 h. Then, ethanol was removed and the mixture was extracted with ethyl acetate and removed the solvent to obtain intermediate **1**. Then chloroacetyl chloride (2.0 mmol) was added to a solution of intermediate **1** (2.0 mmol) in toluene and resulting mixture stirred for 6–8 h at 110–120 °C. After complication of the reaction, the solvent was removed and the residue was recrystallized from ethanol to obtain intermediate **2**. Finally, K_2_CO_3_ (1.0 mmol) was added to a solution of corresponding 1,3,4-oxadiazole/thiadiazole thiol intermediate (1.0 mmol) in MeCN (20 mL) and stirred at room temperature for 0.5 h. Then intermediate **2** (1.0 mmol) was added and the mixture was refluxed. After complication of the reaction, the solvent was removed and the residue was recrystallized from ethanol to obtain the target compounds **5a–5i** with 34.8–62.3% yields.

#### General synthesis procedure for compounds **5j–5r**

**Scheme 2:**
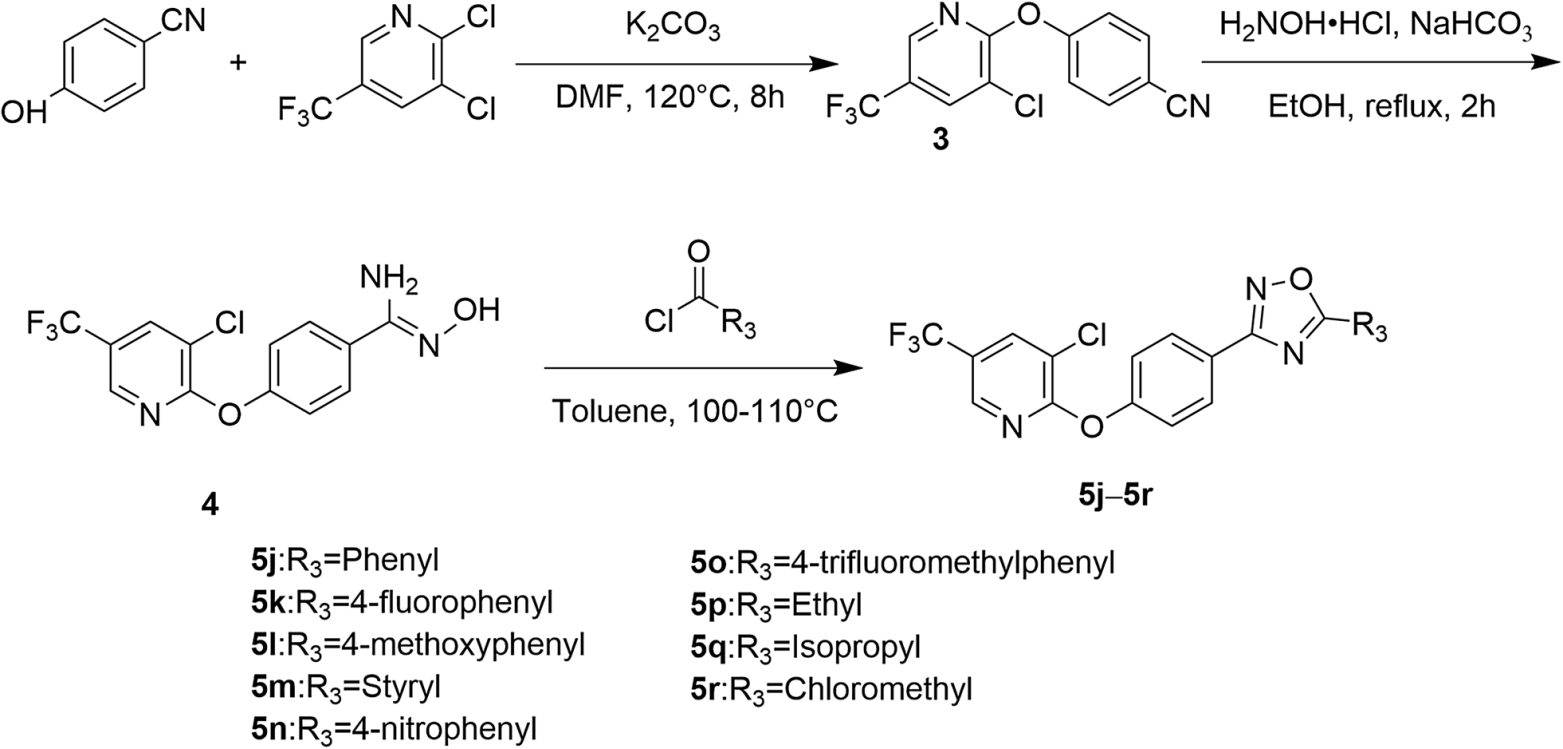
Synthesis of target compounds **5j–5r**

A mixture of 2,3-dichloro-5-(trifluoromethyl) pyridine (5.0 mmol) and 4-hydroxybenzonitrile (5.0 mmol) in DMF (8 mL) was first stirred at room temperature for 0.5 h. Then K_2_CO_3_ (10.0 mmol) was added and the mixture was refluxed for 8 h. After complication of the reaction, the mixture was poured into 100 mL of ethanol. The precipitate was filtered off and to obtain intermediate **3**. Then, a mixture of intermediate **3** (3.0 mmol), NaHCO_3_ (3.0 mmol) and hydroxylamine hydrochloride (4.5 mmol) in ethanol (20 mL) was refluxed for 2 h. The solvent was removed and the residue was poured into water. The mixture was extracted with ethyl acetate and removed the solvent to obtain intermediate **4**. Then substituted acyl chloride (2.0 mmol) was added to a solution of intermediate **4** (2.0 mmol) in toluene and resulting mixture stirred for 6–8 h at 110–120 °C. After complication of the reaction, the solvent was removed and the residue was recrystallized from ethanol to afford the target compounds **5j–5r** with 31.7–62.9% yields (Scheme 2).

#### General synthesis procedure for compounds **5s–5z**

**Scheme 3:**
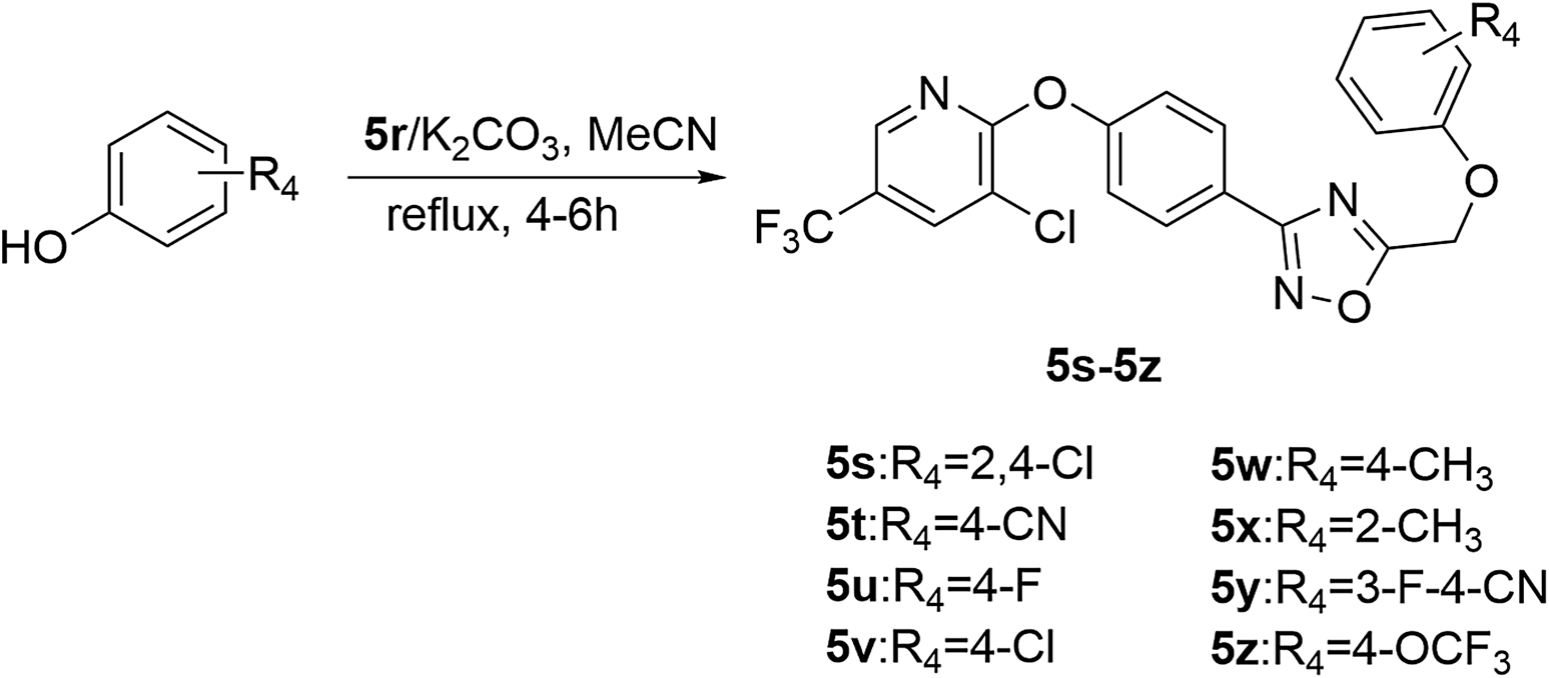
Synthesis of target compounds **5s–5z**

K_2_CO_3_ (1.0 mmol) was added to a solution of substituted phenol (1.0 mmol) in MeCN (20 mL) and stirred at room temperature for 0.5 h. Then compound **5r** (1.0 mmol) was added and the mixture was refluxed for 4–6 h. After complication of the reaction, the solvent was removed and the residue was recrystallized from ethanol to obtain the target compounds **5s–5z** with 41.8–61.8% yields (Scheme 3).

*5*-*(((5*-*(4*-*fluorobenzyl)*-*1,3,4*-*oxadiazol*-*2*-*yl)thio)methyl)*-*3*-*phenyl*-*1,2,4*-*oxadiazole (****5a****)* Yellow solid; yield 55.0%; mp: 106.5–107.1 °C; ^1^H NMR (400 MHz, CDCl_3_) *δ* 7.47 (d, *J* = 7.6 Hz, 3H, Ph-H), 7.47 (d, *J* = 7.6 Hz, 3H, Ph-H) 7.19 (d, *J* = 8.6 Hz, 2H, Ph-H), 4.75 (s, 2H, –CH_2_–). ^13^C NMR (101 MHz, CDCl_3_) *δ* 174.47, 168.81, 165.77, 162.59, 161.69, 131.52, 129.33, 129.33, 128.77, 128.77, 127.49, 127.49, 126.13, 119.59, 116.62, 116.40, 31.44; HRMS (ESI) calcd for C_17_H_12_N_4_O_2_SF [M+H]^+^: 355.06534, found 355.06595.

*5*-*(((5*-*(4*-*chlorophenyl)*-*1,3,4*-*oxadiazol*-*2*-*yl)thio)methyl)*-*3*-*(4*-*fluorophenyl)*-*1,2,4*-*oxadiazole (****5b****)* Yellow solid; yield 45.0%; mp: 101.9–103.2 °C; ^1^H NMR (400 MHz, CDCl_3_) *δ* 8.05 (dd, *J* = 8.9, 5.4 Hz, 2H, Ph-H), 7.94 (d, *J* = 8.7 Hz, 2H, Ph-H), 7.48 (d, *J* = 8.7 Hz, 2H, Ph-H), 7.15 (t, *J* = 8.7 Hz, 2H, Ph-H), 4.75 (s, 2H, –CH_2_–). ^13^C NMR (101 MHz, CDCl_3_) *δ* 174.96, 169.70, 168.77, 162.58 161.73, 131.42, 129.57, 129.57, 129.29, 129.29, 128.89, 128.89, 127.86, 127.86, 127.54, 126.32, 31.46; HRMS (ESI) calcd for C_17_H_11_N_4_O_2_SFCl [M+H]^+^: 389.02698, found 353.02681.

*5*-*(((5*-*(4*-*chlorobenzyl)*-*1,3,4*-*oxadiazol*-*2*-*yl)thio)methyl)*-*3*-*phenyl*-*1,2,4*-*oxadiazole (****5c****)* Brown solid; yield 57.3%; mp: 83.5–84.7 °C; ^1^H NMR (400 MHz, CDCl_3_) *δ* 8.06–8.02 (m, 2H, Ph-H), 7.51–7.45 (m, 3H, Ph-H), 7.32–7.27 (m, 2H, Ph-H), 7.22 (t, *J* = 6.8 Hz, 2H, Ph-H), 4.78 (s, 2H, –CH_2_–), 4.35 (s, 2H, –CH_2_–). ^13^C NMR (101 MHz, CDCl_3_) *δ* 174.85, 170.43, 168.70, 162.95, 134.90, 133.67, 131.43, 130.15, 130.15, 129.25, 129.25, 128.88, 128.88, 127.48, 127.48, 126.27, 35.80, 27.65; HRMS (ESI) calcd for C_18_H_14_N_4_OS_2_Cl [M+H]^+^: 401.02856, found 401.02921.

*5*-*(((5*-*(4*-*fluorophenyl)*-*1,3,4*-*thiadiazol*-*2*-*yl)thio)methyl)*-*3*-*(o*-*tolyl)*-*1,2,4*-*oxadiazole (****5d****)* Yellow solid; yield 48.6%; mp: 98.1–98.7 °C; ^1^H NMR (500 MHz, CDCl_3_) *δ* 7.97–7.92 (m, 3H, Ph-H), 7.47 (d, *J* = 8.7 Hz, 2H, Ph-H), 7.40–7.36 (m, 1H, Ph-H), 7.32–7.26 (m, 2H, Ph-H), 4.76 (s, 2H, –CH_2_–), 2.59 (s, 3H, –CH_3_). ^13^C NMR (126 MHz, CDCl_3_) *δ* 173.38, 169.47, 165.86, 162.06, 138.42, 131.54, 130.97, 130.22, 130.22, 129.63, 129.63, 128.16, 126.10, 125.47, 121.83, 114.02, 26.85, 22.28; HRMS (ESI) calcd for C_18_H_15_N_4_OS_2_ [M+H]^+^: 367.06766, found 367.06818.

*5*-*(((5*-*(3*-*chlorobenzyl)*-*1,3,4*-*oxadiazol*-*2*-*yl)thio)methyl)*-*3*-*(o*-*tolyl)*-*1,2,4*-*oxadiazole (****5e****)* Yellow solid; yield 62.3%; mp: 82.6–83.2 °C; ^1^H NMR (500 MHz, CDCl_3_) *δ* 7.94–7.91 (m, 1H, Ph-H), 7.39 (td, *J* = 7.6, 1.4 Hz, 1H, Ph-H), 7.32–7.26 (m, 4H, Ph-H), 7.23–7.19 (m, 2H, Ph-H), 4.67 (s, 2H, –CH_2_–), 4.15 (s, 2H, –CH_2_–), 2.58 (s, 3H, –CH_3_). ^13^C NMR (126 MHz, CDCl_3_) *δ* 173.34, 169.40, 166.83, 162.52, 138.43, 133.84, 131.76, 131.55, 131.55, 130.97, 130.24, 130.24, 129.25, 129.25, 126.12, 125.47, 31.35, 26.69, 22.28; HRMS (ESI) calcd for C_19_H_16_N_4_O_2_SCl [M+H]^+^: 399.06714, found 399.06770.

*3*-*(4*-*chlorophenyl)*-*5*-*(((5*-*(4*-*fluorophenyl)*-*1,3,4*-*oxadiazol*-*2*-*yl)thio)methyl)*-*1,2,4*-*oxadiazol*e *(****5f****)* White solid; yield 60.2%; mp: 106.6–108.1 °C; ^1^H NMR (500 MHz, CDCl_3_) *δ* 8.03–7.97 (m, 4H, Ph-H), 7.46–7.42 (m, 2H, Ph-H), 7.21–7.16 (m, 2H, Ph-H), 4.74 (s, 2H, –CH_2_–). ^13^C NMR (126 MHz, CDCl_3_) *δ* 174.75, 168.15, 165.95, 161.70, 137.79, 130.03, 130.03, 129.59, 129.59, 129.01, 129.01, 128.91, 128.91, 124.74, 119.65, 116.72, 26.83; HRMS (ESI) calcd for C_17_H_11_N_4_O_2_SFCl [M+H] + : 389.02637, found 389.02698.

*5*-*(((5*-*(4*-*chlorobenzyl)*-*1,3,4*-*oxadiazol*-*2*-*yl)thio)methyl)*-*3*-*(4*-*chlorophenyl)*-*1,2,4*-*oxadiazole (****5*** ***g****)* Yellow solid; yield 56.3%; mp: 84.7–86.0 °C; ^1^H NMR (500 MHz, CDCl_3_) *δ* 7.99–7.95 (m, 2H, Ph-H) 7.46–7.43 (m, 2H, Ph-H), 7.32–7.28 (m, 2H, Ph-H), 7.22–7.19 (m, 2H, Ph-H), 4.77 (s, 2H, –CH_2_–), 4.34 (s, 2H, –CH_2_–). ^13^C NMR (126 MHz, CDCl_3_) *δ* 175.22, 170.54, 168.02, 162.96, 137.69, 134.97, 133.80, 130.26, 130.26, 129.59, 129.59, 129.14, 129.14, 128.89, 128.89, 124.88, 35.90, 27.63; HRMS (ESI) calcd for C_18_H_13_N_4_O_2_SCl_2_ [M+H]^+^: 419.01233, found 419.01308.

*5*-*(((5*-*(4*-*chlorobenzyl)*-*1,3,4*-*thiadiazol*-*2*-*yl)thio)methyl)*-*3*-*(4*-*chlorophenyl)*-*1,2,4*-*oxadiazole (****5h****)* Yellow solid; yield 34.8%; mp: 83.0–84.3 °C; ^1^H NMR (500 MHz, CDCl_3_) *δ* 7.99–7.93 (m, 2H, Ph-H), 7.48–7.40 (m, 2H, Ph-H), 7.31–7.26 (m, 2H, Ph-H), 7.21 (d, *J* = 8.5 Hz, 2H, Ph-H), 4.65 (s, 2H, –CH_2_–), 4.15 (s, 2H, -CH_2_-). ^13^C NMR (126 MHz, CDCl_3_) *δ* 174.67, 168.08, 166.88, 162.41, 137.80, 133.87, 131.71, 130.39, 130.39, 129.61, 129.61, 129.30, 129.30, 129.00, 128.77, 124.71, 31.44, 26.67; HRMS (ESI) calcd for C_18_H_13_N_4_OS_2_Cl_2_ [M+H]^+^: 434.98953, found 434.99023.

*5*-*(((5*-*(4*-*chlorophenyl)*-*1,3,4*-*thiadiazol*-*2*-*yl)thio)methyl)*-*3*-*(p*-*tolyl)*-*1,2,4*-*oxadiazole (****5i****)* Yellow solid; yield 41.5%; mp: 106.7–107.8 °C; ^1^H NMR (400 MHz, CDCl_3_) *δ* 7.97–7.92 (m, 4H, Ph-H), 7.47 (d, *J* = 8.6 Hz, 2H, Ph-H), 7.27 (d, *J* = 8.2 Hz, 2H, Ph-H), 4.74 (s, 2H, –CH_2_–), 2.41 (s, 3H, –CH_3_). ^13^C NMR (101 MHz, CDCl_3_) *δ* 174.16, 165.79, 161.95, 141.94, 138.31, 133.80, 129.52, 129.52, 129.59, 129.59, 128.00, 128.00, 127.44, 127.44, 123.32 121.75, 26.82, 21.62; HRMS (ESI) calcd for C_18_H_15_N_4_OS_2_ [M+H]^+^: 367.06763, found 367.06818.

*3*-*(4*-*((3*-*chloro*-*5*-*(trifluoromethyl)pyridin*-*2*-*yl)oxy)phenyl)*-*5*-*phenyl*-*1,2,4*-*oxadiazole (****5j****)* White solid; yield 31.7%; mp: 94.5–95.0 °C; ^1^H NMR (400 MHz, DMSO-*d*_6_) *δ* 8.63 (d, *J* = 2.2 Hz, 1H, Pyridine-H), 8.56 (dd, *J* = 2.1, 0.9 Hz, 1H, Pyridine-H), 8.23–8.17 (m, 4H, Ph-H), 7.79–7.74 (m, 1H, Ph-H), 7.71–7.66 (m, 2H, Ph-H), 7.52–7.48 (m, 2H, Ph-H). ^13^C NMR (101 MHz, DMSO-*d*_6_) *δ* 175.99, 168.17, 160.92, 155.64, 143.64, 137.85, 133.92, 130.09, 130.09, 129.36, 129.36, 128.43, 124.79, 124.06, 123.81, 123.09, 123.09, 122.52, 122.14, 119.16; HRMS (ESI) calcd for C_20_H_12_N_3_O_2_F_3_Cl [M+H]^+^: 418.05539, found 418.05647.

*3*-*(4*-*((3*-*chloro*-*5*-*(trifluoromethyl)pyridin*-*2*-*yl)oxy)phenyl)*-*5*-*(4*-*fluorophenyl)*-*1,2,4*-*oxadiazole (****5k****)* White solid; yield 41.8%; mp: 106.5–107.3 °C; ^1^H NMR (400 MHz, DMSO-*d*_6_) *δ* 8.64 (d, *J* = 2.1 Hz, 1H, Pyridine-H), 8.56 (dd, *J* = 2.1, 0.9 Hz, 1H, Pyridine-H), 8.23–8.17 (m, 4H, Ph-H), 7.79–7.74 (m, 1H, Ph-H), 7.78–7.74 (m, 2H, Ph-H), 7.54–7.47 (m, 2H, Ph-H). ^13^C NMR (101 MHz, DMSO-*d*_6_) *δ* 175.15, 168.22, 160.91, 155.69, 143.62, 138.76, 137.89, 130.28, 130.28, 129.37, 129.37, 124.79, 124.05, 123.91, 123.11, 123.11, 122.60, 122.19 119.16, 119.16; HRMS (ESI) calcd for C_20_H_11_N_3_O_2_F_4_Cl [M+H]^+^: 436.04312, found 436.04704.

*3*-*(4*-*((3*-*chloro*-*5*-*(trifluoromethyl)pyridin*-*2*-*yl)oxy)phenyl)*-*5*-*(4*-*methoxyphenyl)*-*1,2,4*-*oxadiazole (****5l****)* Yellow solid; yield 62.9%; mp: 95.2–96.0 °C; ^1^H NMR (400 MHz, DMSO-*d*_6_) *δ* 8.69 (d, *J* = 2.0 Hz, 1H, Pyridine-H), 8.61 (d, *J* = 1.1 Hz, 1H, Pyridine-H), 8.25–8.19 (m, 4H, Ph-H), 7.57–7.53 (m, 2H, Ph-H), 7.27 (d, *J* = 8.9 Hz, 2H, Ph-H), 3.95 (s, 3H, –OCH_3_). ^13^C NMR (101 MHz, DMSO-*d*_6_) *δ* 175.83, 167.99, 160.94, 155.53, 143.62, 137.88, 130.47, 130.47, 129.31, 129.31, 124.79, 124.21, 123.07, 123.07, 122.48, 122.15, 119.12, 116.11, 115.51, 115.51, 56.49; HRMS(ESI) calcd for C_21_H_14_N_3_O_3_F_3_Cl; [M+H]^+^: 448.06703, found 448.06580.

*(E)*-*3*-*(4*-*((3*-*chloro*-*5*-*(trifluoromethyl)pyridin*-*2*-*yl)oxy)phenyl)*-*5*-*styryl*-*1,2,4*-*oxadiazole (****5*** ***m****)* Light yellow solid; yield 41.7%; mp: 121.0–121.5 °C; ^1^H NMR (400 MHz, DMSO-*d*_6_) *δ* 8.64 (d, *J* = 2.1 Hz, 1H, Pyridine-H), 8.56 (dd, *J* = 2.1, 1.0 Hz, 1H, Pyridine-H), 8.14–8.09 (m, 2H, –CH–), 7.98 (d, *J* = 16.4 Hz, 1H, Ph-H), 7.90–7.86 (m, 2H Ph-H), 7.52–7.46 (m, 6H, Ph-H). ^13^C NMR (101 MHz, DMSO-*d*_6_) *δ* 175.99, 167.89, 160.93, 155.54, 144.29, 143.34, 137.88, 134.69, 131.20, 129.51, 129.51, 129.26, 129.26, 128.92, 124.79, 124.19, 123.07, 123.07, 122.48, 122.12, 119.14, 110.66; HRMS (ESI) calcd for C_22_H_14_N_3_O_2_F_3_Cl [M+H]^+^: 444.07135, found 444.07212.

*3*-*(4*-*((3*-*chloro*-*5*-*(trifluoromethyl)pyridin*-*2*-*yl)oxy)phenyl)*-*5*-*(4*-*nitrophenyl)*-*1,2,4*-*oxadiazole (****5n****)* White solid; yield 35.8%; mp: 182.4–183.7 °C; ^1^H NMR (400 MHz, DMSO-*d*_6_) *δ* 8.64 (d, *J* = 2.1 Hz, 1H, Pyridine-H), 8.65 (d, *J* = 1.9 Hz, 1H, Pyridine-H), 8.49 (d, *J* = 3.4 Hz, 4H, Ph-H), 8.24–8.19 (m, 2H, Ph-H), 7.56–7.50 (m, 2H, Ph-H). ^13^C NMR (101 MHz, DMSO-*d*_6_) *δ* 175.1, 168.14, 160.61, 157.93, 155.62, 143.61, 137.82, 132.66, 131.32, 129.75, 129.33, 129.33, 124.78, 123.90, 123.90, 122.50, 121.84, 120.51, 119.14, 117.25; HRMS (ESI) calcd for C_20_H_11_N_4_O_4_F_3_Cl [M+H]^+^: 463.04154, found 463.04025.

*3*-*(4*-*((3*-*chloro*-*5*-*(trifluoromethyl)pyridin*-*2*-*yl)oxy)phenyl)*-*5*-*(4*-*(trifluoromethyl)phenyl)*-*1,2,4*-*oxadiazole (****5o****)* White solid; yield 44.3%; mp: 102.8–103.7 °C; ^1^H NMR (400 MHz, DMSO-*d*_6_) *δ* 8.59 (d, *J* = 2.2 Hz, 1H, Pyridine-H), 8.51 (d, *J* = 1.0 Hz, 1H, Pyridine-H), 8.36 (d, *J* = 8.3 Hz, 2H, Pyridine-H) 8.17–8.13 (m, 2H, Ph-H), 8.00 (d, *J* = 8.4 Hz, 2H, Ph-H), 7.48–7.45 (m, 2H, Ph-H). ^13^C NMR (101 MHz, DMSO-*d*_6_) *δ* 174.84, 168.40, 160.95, 155.78, 143.68, 137.93, 133.62, 133.36, 133.11, 129.87, 129.87, 128.32, 127.61, 125.24, 124.57, 123.81, 123.81, 122.73, 122.21, 120.00, 119.22; HRMS (ESI) calcd for C_21_H_11_N_3_O_2_F_6_Cl; [M+H]^+^: 486.04385, found 486.04257.

*3*-*(4*-*((3*-*chloro*-*5*-*(trifluoromethyl)pyridin*-*2*-*yl)oxy)phenyl)*-*5*-*isopropyl*-*1,2,4*-*oxadiazole (****5p****)* White solid; yield 56.1%; mp 88.5–89.2 °C; ^1^H NMR (400 MHz, DMSO-*d*_6_) *δ* 8.63 (d, *J* = 2.1 Hz, 1H, Pyridine-H), 8.55–8.54 (m, 1H, Pyridine-H), 8.09 (d, *J* = 8.8 Hz, 2H, Ph-H), 7.47–7.43 (m, 2H, Ph-H), 2.81 (d, *J* = 63.7 Hz, 1H, –CH–), 1.39 (d, *J* = 7.0 Hz, 6H, –CH_3_). ^13^C NMR (101 MHz, DMSO-*d*_6_) *δ* 184.70, 167.30, 160.89, 155.43, 143.63, 137.83, 129.75, 129.19, 126.50, 124.78, 124.22, 123.01, 122.48, 122.11, 119.13, 27.28, 20.29; HRMS (ESI) calcd for C_17_H_14_N_3_O_2_F_3_Cl [M+H]^+^: 384.07212, found 384.07095.

*3*-*(4*-*((3*-*chloro*-*5*-*(trifluoromethyl)pyridin*-*2*-*yl)oxy)phenyl)*-*5*-*ethyl*-*1,2,4*-*oxadiazole (****5q****)* White solid; yield 44.8%; mp 83.3–84.0 °C; ^1^H NMR (400 MHz, DMSO-*d*_6_) *δ* 8.63 (d, *J* = 2.0 Hz, 1H, Pyridine-H), 8.55 (d, *J* = 1.1 Hz, 1H, Pyridine-H), 8.09 (d, *J* = 8.7 Hz, 2H, Ph-H), 7.45 (d, *J* = 8.7 Hz, 2H, Ph-H), 3.03 (d, *J* = 7.6 Hz, 2H, –CH_2_–), 1.35 (t, *J* = 7.6 Hz, 3H, –CH_3_–). ^13^C NMR (101 MHz, DMSO-*d*_6_) *δ* 181.81, 167.34, 160.91, 155.42, 143.62, 137.84, 129.77, 129.15, 124.79, 124.23, 123.02, 122.46, 122.10, 119.12, 20.07, 10.92; HRMS (ESI) calcd for C_16_H_12_N_3_O_2_F_3_Cl [M+H]^+^: 370.05647, found 370.05554.

*3*-*(4*-*((3*-*chloro*-*5*-*(trifluoromethyl)pyridin*-*2*-*yl)oxy)phenyl)*-*5*-*(chloromethyl)*-*1,2,4*-*oxadiazole (****5r****)* White solid; yield 46.7%; mp: 94.6–95.7 °C; ^1^H NMR (500 MHz, DMSO-*d*_6_) *δ* 8.59 (d, *J* = 2.1 Hz, 1H, Pyridine-H), 8.51 (dd, *J* = 1.9, 0.8 Hz, 1H, Pyridine-H), 8.10–8.05 (m, 2H, Ph-H), 7.46–7.42 (m, 2H, Ph-H), 5.17 (s, 2H, –CH_2_–). ^13^C NMR (126 MHz, DMSO-*d*_6_) *δ* 176.24, 167.98, 160.93, 155.82, 143.69, 138.04, 137.82, 129.39, 124.57, 123.58, 123.41, 122.05, 120.00, 119.23, 34.26; HRMS (ESI) calcd for C_15_H_9_N_3_O_2_F_3_Cl_2_ [M+H]^+^: 390.00184, found 390.00082.

*3*-*(4*-*((3*-*chloro*-*5*-*(trifluoromethyl)pyridin*-*2*-*yl)oxy)phenyl)*-*5*-*((2,4*-*dichlorophenoxy)methyl)*-*1,2,4*-*oxadiazole (****5*** ***s****)* Yellow solid; yield 49.0%; mp: 97.1–98.0 °C; ^1^H NMR (400 MHz, DMSO-*d*_6_) *δ* 8.59 (d, *J* = 2.2 Hz, 1H, Pyridine-H), 8.50 (s, 1H, Pyridine-H), 8.06 (d, *J* = 8.7 Hz, 2H, Ph-H), 7.62 (d, *J* = 2.5 Hz, 1H, Ph-H), 7.45–7.42 (m, 2H, Ph-H), 7.40 (dd, *J* = 8.9, 2.6 Hz, 1H, Ph-H), 7.32 (d, *J* = 9.0 Hz, 1H, Ph-H), 5.72 (s, 2H, –CH_2_–). ^13^C NMR (101 MHz, DMSO-*d*_6_) *δ* 175.82, 167.71, 160.94, 155.77, 152.45, 152.27, 143.67, 137.92, 130.19, 130.19, 128.78, 126.52, 124.57, 123.64, 123.63, 123.63, 122.67, 122.20, 119.21, 116.34, 62.50; HRMS (ESI) calcd for C_21_H_12_N_3_O_3_F_3_Cl_3_ [M+H]^+^: 515.98779, found 515.98909.

*4*-*((3*-*(4*-*((3*-*chloro*-*5*-*(trifluoromethyl)pyridin*-*2*-*yl)oxy)phenyl)*-*1,2,4*-*oxadiazol*-*5*-*yl)methoxy)benzonitrile (****5t****)* White solid; yield 61.8%; mp: 119.0–120.2 °C; ^1^H NMR (400 MHz, DMSO-*d*_6_) *δ*: 8.59 (d, *J* = 2.1 Hz, 1H, Pyridine-H), 8.50 (d, *J *= 1.1 Hz, 1H, Pyridine-H), 8.06 (d, *J* = 8.7 Hz, 2H, Ph-H), 7.82 (d, *J* = 8.9 Hz, 2H, Ph-H), 7.44 (d, *J* = 8.7 Hz, 2H, Ph-H), 7.26 (d, *J* = 8.9 Hz, 2H, Ph-H), 5.72 (s, 2H, –CH_2_–). ^13^C NMR (101 MHz, DMSO-*d*_6_) *δ* 175.81, 167.72, 160.93, 155.78, 143.68, 137.95, 134.94, 134.94, 129.42, 129.42, 124.57, 123.62, 123.22, 123.22, 122.63, 122.21, 119.39, 119.22, 116.54, 116.36, 104.80, 61.61; HRMS (ESI) calcd for C_22_H_13_N_4_O_3_F_3_Cl [M+H]^+^: 473.06228, found 473.06107.

*3*-*(4*-*((3*-*chloro*-*5*-*(trifluoromethyl)pyridin*-*2*-*yl)oxy)phenyl)*-*5*-*((4*-*fluorophenoxy)methyl)*-*1,2,4*-*oxadiazole (****5u****)* Yellow solid; yield 41.8%; mp: 101.1–102.0 °C; ^1^H NMR (400 MHz, DMSO-*d*_6_) *δ* 8.63 (d, *J* = 2.1 Hz, 1H, Pyridine-H), 8.55–8.53 (m, 1H, Pyridine-H), 8.13–8.09 (m, 2H, Ph-H), 7.49–7.45 (m, 2H, Ph-H), 7.21–7.11 (m, 4H, Ph-H), 5.60 (s, 2H, –CH_2_–). ^13^C NMR (101 MHz, DMSO-*d*_6_) *δ* 176.30, 167.61, 160.88, 158.86, 156.50, 155.69, 154.06, 143.61, 137.85, 129.34, 129.34, 124.78, 123.65, 123.14, 123.14, 122.52, 122.13, 119.16, 116.99, 116.99, 61.91; HRMS (ESI) calcd for C_21_H_13_N_3_O_3_F_4_Cl [M+H]^+^: 466.05761, found 466.05652.

*3*-*(4*-*((3*-*chloro*-*5*-*(trifluoromethyl)pyridin*-*2*-*yl)oxy)phenyl)*-*5*-*((4*-*chlorophenoxy)methyl)*-*1,2,4*-*oxadiazole (****5v****)* Brown solid; yield 48.5%; mp: 92.3–93.1 °C; ^1^H NMR (400 MHz, DMSO-*d*_6_) *δ* 8.64 (d, *J *= 2.0 Hz, 1H, Pyridine-H), 8.55 (dd, *J* = 2.1, 0.9 Hz, 1H, Pyridine-H), 8.12–8.09 (m, 2H, Ph-H), 7.49–7.46 (m, 2H, Ph-H), 7.42–7.39 (m, 2H, Ph-H), 7.16–7.13 (m, 2H, Ph-H), 5.64 (s, 2H, –CH_2_–). ^13^C NMR (101 MHz, DMSO-*d*_6_) *δ* 176.16, 167.62, 160.88, 156.59, 155.70, 143.63, 137.87, 129.95, 129.95, 129.35, 126.11, 124.79, 123.62, 123.17, 123.17, 122.50, 122.13, 119.15, 117.13, 117.13, 61.59; HRMS (ESI) calcd for C_21_H_13_N_3_O_3_F_3_Cl_2_ [M+H]^+^: 482.02806, found 482.02698.

*3*-*(4*-*((3*-*chloro*-*5*-*(trifluoromethyl)pyridin*-*2*-*yl)oxy)phenyl)*-*5*-*((p*-*tolyloxy)methyl)*-*1,2,4*-*oxadiazole (****5w****)* Yellow solid; yield 54.9%; mp: 84.2–84.7 °C; ^1^H NMR (500 MHz, DMSO-*d*_6_) *δ* 8.59 (d, *J* = 2.1 Hz, 1H, Pyridine-H), 8.50 (dd, *J* = 1.9, 0.8 Hz, 1H, Pyridine-H), 8.09–8.05 (m, 2H, Ph-H), 7.45–7.41 (m, 2H, Ph-H), 7.10 (d, *J* = 8.2 Hz, 2H, Ph-H), 6.96–6.93 (m, 2H, Ph-H), 5.53 (s, 2H, –CH_2_–), 2.20 (s, 3H, –CH_3_). ^13^C NMR (126 MHz, DMSO-*d*_6_) *δ* 176.62, 167.65, 160.94, 155.72, 143.68, 137.94, 131.22, 130.55, 130.55, 129.40, 129.40, 124.57, 123.74, 123.21, 123.21, 122.62, 122.2, 119.22, 115.18, 115.18, 61.39, 20.61; HRMS (ESI) calcd for C_22_H_16_N_3_O_3_F_3_Cl; [M+H]^+^: 462.08268, found 462.08160.

*4*-*(4*-*((3*-*chloro*-*5*-*(trifluoromethyl)pyridin*-*2*-*yl)oxy)phenyl)*-*5*-*((o*-*tolyloxy)methyl)*-*1,2,4*-*oxadiazole (****5x****)* Yellow solid; yield 42.2%; mp: 80.0–80.8 °C; ^1^H NMR (500 MHz, DMSO-*d*_6_) *δ* 8.60 (d, *J* = 2.0 Hz, 1H, Pyridine-H), 8.51 (s, 1H, Pyridine-H), 7.44 (d, *J* = 8.7 Hz, 3H, Ph-H), 7.16 (dd, *J* = 13.7, 7.4 Hz, 2H, Ph-H), 7.05 (d, *J* = 8.0 Hz, 1H, Ph-H), 6.89 (t, *J* = 7.3 Hz, 1H, Ph-H), 5.59 (s, 2H, –CH_2_–), 2.20 (s, 3H, -CH_3_). ^13^C NMR (126 MHz, DMSO-*d*_6_) *δ* 176.70, 167.67, 160.95, 155.98, 155.75, 143.69, 137.95, 131.37, 129.40, 129.40, 127.62, 126.80, 124.58, 123.75, 123.23, 123.23, 122.67, 121.90, 119.21, 112.57, 61.68, 16.45; HRMS (ESI) calcd for C_22_H_16_N_3_O_3_F_3_Cl [M+H]^+^: 462.08268, found 462.08160.

*4*-*((3*-*(4*-*((3*-*chloro*-*5*-*(trifluoromethyl)pyridin*-*2*-*yl)oxy)phenyl)*-*1,2,4*-*oxadiazol*-*5*-*yl)methoxy)*-*2*-*fluorobenzonitrile (****5y****)* Red solid; yield 43.7%; mp: 114.7–115.3 °C; ^1^H NMR (400 MHz, DMSO-*d*_6_) *δ* 8.59 (d, *J* = 2.2 Hz, 1H, Pyridine-H), 8.50 (d, *J* = 1.0 Hz, 1H, Pyridine-H), 8.08–8.04 (m, 2H, Ph-H), 7.92–7.87 (m, 1H, Ph-H), 7.45–7.42 (m, 2H, Ph-H), 7.38 (dd, *J* = 11.6, 2.4 Hz, 1H, Ph-H), 7.14 (dd, *J* = 8.8, 2.4 Hz, 1H, Ph-H), 5.75 (s, 2H, –CH_2_–). ^13^C NMR (101 MHz, DMSO-*d*_6_) *δ* 175.34, 167.65, 160.87, 155.72, 143.61, 137.87, 135.49, 129.36, 129.36, 124.77, 123.58, 123.35, 123.35, 122.07, 119.15, 114.63, 113.14, 104.17, 104.03, 103.59, 93.50, 62.12; HRMS (ESI) calcd for C_22_H_12_N_4_O_3_F_4_Cl [M+H]^+^: 491.05286, found 491.05167.

*3*-*(4*-*((3*-*chloro*-*5*-*(trifluoromethyl)pyridin*-*2*-*yl)oxy)phenyl)*-*5*-*ethyl*-*1,2,4*-*oxadiazole (****5z****)* White solid; yield 57.5%; mp: 105.2–106.7 °C; ^1^H NMR (400 MHz, DMSO-*d*_6_) *δ* 8.64 (d, *J* = 2.0 Hz, 1H, Pyridine-H), 8.55 (d, *J* = 1.1 Hz, 1H, Pyridine-H), 8.54 (dd, *J* = 2.1, 0.9 Hz, 2H, Ph-H), 8.13–8.09 (m, 2H, Ph-H), 7.50–7.45 (m, 2H, Ph-H), 7.25–7.19 (m, 2H, Ph-H), 5.67 (s, 2H, –CH_2_–). ^13^C NMR (101 MHz, DMSO-*d*_6_) *δ* 176.12, 167.63, 160.88, 156.57, 155.70, 143.62, 143.11, 137.89, 137.87, 129.36, 129.36, 124.78, 123.62, 123.20, 123.20, 122.51, 122.13, 121.87, 119.24, 116.68, 116.68, 61.75; HRMS (ESI) calcd for C_22_H_13_N_3_O_4_F_6_Cl [M+H]^+^: 532.04933, found 532.04822.

### Nematocidal activity

The nematocidal activity of the target compound was carried out according to the reported method [33]. The tomato grown in the greenhouse for cultivating southern root-knot nematodes were uprooted and washed with water. Then take the eggs of the roots with a toothpick and place them in a petri dish containing distilled water. Second instar larvae were collected after 3–7 days of incubation at 27 °C. All tested compounds were dissolved in DMF and diluted with 1% Tween 80 (final concentration of DMF was 0.5%). 250 μL of the test solution was added to a 48-well biochemical culture dish and tested. Subsequently, approximately 100 nematodes were added to each well. Abamectin was used as a positive control, and a test solution containing no compound was used as a negative control. After 48 h of treatment with the compound, the nematode was transferred to clear water for resuscitation, and the nematode that did not move was considered dead.

$$\begin{aligned} {\text{Corrected mortality }}\% \, = & \, [\left( {{\text{mortality of treatment }}\% \, - {\text{ mortality of negative control }}\% } \right) \\ /\left( { 1 { } - {\text{ mortality of negative control }}\% } \right)] \, \times { 1}00 \\ \end{aligned}$$

### Antifungal assay

The mycelium growth rate method was utilized to evaluate in vitro antifungal activities of target compounds against *R. solani* [34]. The DMSO solution of the test compound was added to a sterilized petri dish containing about 10 mL of molten potato dextrose agar (PDA). Subsequently, a mycelial plug with a diameter of 4 mm was cut from the fungal colony and placed in the center of the PDA plate at 28 ± 1 °C for 4 days. For each compound, antifungal assays were performed in triplicate. In addition, pure DMSO and commercial fungicide (Hymexazol) were also used as negative and positive control agents, respectively.

The inhibition rate (*I*) of the tested compound was determined based on the following formula:

$$I = \left( {{\text{C}} - {\text{T}}} \right)/\left( {{\text{C}} - 0. 4} \right) \times 100\%$$

In the formula, C represents the average mycelial diameter of negative control and T represents the average mycelial diameter of tested compound-treated PDA.

### Antibacterial activity in vitro

The previously described method was used for in vitro antibacterial activity testing [35–37]. The 50 μL culture of *Xoo* or *Xoc* in logarithmic growth phase were added to the test tubes with 5 mL of NB medium containing different concentrations of the target compound, respectively. The commercial bacteriacide thiodiazole copper (TDC, 20% suspending agent) and bismerthiazol (BMT, 20% wettable powder) as positive controls, while the same treatment without compound as the negative control. Then, the absorbance at 600 nm was measured when the tubes were inoculated at 28 °C for 48 h with shaking at 180 rpm. The inhibition rate (the inhibition rate refers to the proportion of bacteria whose growth was inhibited) was calculated by the following equation: where the absorbance of the negative control group was expressed as OD_CK_, and the absorbance of the treated group was expressed as OD_T_.

$${\text{Inhibition rate }}\left( \% \right) \, = \, \left( {OD_{\text{CK}} - OD_{\text{T}} } \right) /OD_{\text{CK}} \times { 1}00$$

### Antibacterial activity in vivo

In vivo biometric against rice bacterial leaf blight. The curative and protection activities of compound **5v** against rice bacterial leaf blight were determined by Schaad’s method with some slight modifications [4]. The curative activity of the rice plant bacterial leaf blight-reducing compound **5v** in potted plants was determined under controlled conditions in the growth room. About 8 weeks after planting the “Fengyouxiangzhan” rice seeds, *Xoo* was inoculated on the rice leaves. One day after the inoculation, 200 μg/mL **5v** solutions was evenly sprayed onto the rice leaves until dripping, and 200 μg/mL BMT and TDC solutions, and distilled water was evenly sprayed as positive and negative control groups, respectively. Then, all inoculated rice plants were placed in a plant growth chamber (28 °C and 90% relative humidity). On the 14th day after spraying, the disease index of the inoculated rice leaves was measured. Similarly, the protective activity of reducing rice bacterial leaf blight of compound **5v** was also evaluated, the difference is that 1 day after spraying the compound solution and distilled water, *Xoo* was inoculated on rice leaves and the disease index of the inoculated rice leaves was measured on the 14th day after inoculation. First, measure the spot area of each leaf and the entire leaf area, and then calculate the percentage of the spot area in the entire leaf area. Secondly, these leaves were classed according to the following grading standards. Grade 1: the area of disease spot accounts for less than 5% of the whole leaf area; Grade 3: the area of disease spot accounts for 6–10% of the whole leaf area; Grade 5: the area of disease spot accounts for 11–20% of the whole leaf area; Grade 7: the area of disease spot 6 accounts for 21–50% of the whole leaf area; Grade 9: the area of disease spot accounts for more than 50% of the whole leaf area. Finally, the disease index (C or T) was calculated using the following formula: Disease index (C or T) = ∑ (the number of leaves at each Grade × the corresponding Grade)/(the total number of leaves × the superlative Grade). The control coefficients I (%) for the curative and protection activities are calculated by the following equation. In the equation, C is the disease index of the negative control and T is the disease index of the treatment group.

$${\text{Control efficiency I }}\left( \% \right) = \left( {{\text{C }} - {\text{ T}}} \right)/{\text{C }} \times 100$$

## Results and discussion

### Design of novel 1,2,4-oxadiazole derivatives

1,2,4-Oxadiazole heterocycle is an important pharmacophore to design novel drug molecules. The compounds containing 1,2,4-oxadiazole skeleton possess various bioactivity in agricultural, including antibacterial, antifungal and nematocidal activities. Of which, tioxazafen is a new nematicide with unique mechanism of action developed by Monsanto. In our previous works, some 1,3,4-thiadiazol or 1,3,4-oxadiazole derivatives were designed and synthesized, and they exhibited good antibacterial, antifungal and nematocidal activities. So, we firstly introduced 1,3,4-thiadiazol or 1,3,4-oxadiazole into 1,2,4-oxadiazole skeleton to find highly active compounds. Meanwhile, the literature survey reveals that fluopyram showed good antibacterial activity and nematocidal activity, and the important pharmacophore is trifluoromethyl pyridine moiety. Encouraged by this results, we designed the novel 1,2,4-oxadiazole derivatives containing a trifluoromethyl pyridine moiety to find new lead compounds.

### Chemistry

^1^H NMR, ^13^C NMR, and HRMS were used to characterize the physical properties of the target compounds **5a**–**5z**. ^1^H NMR, ^13^C NMR, and HRMS data are provided in Additional file 1. In ^1^H NMR spectra of compound **5v**, singlet at *δ* 8.64–8.55 ppm reveals the presence of Pyridine-H protons, singlet at *δ* 8.12–7.13 ppm reveals the presence of Ph-H protons. From the analysis of the ^13^C NMR spectrum of the compound **5v**, it can be seen that 176.16 and 167.62 ppm are the absorption peaks of carbon on oxadiazole structure, 160.88, 156.59 and 155.70 ppm are the absorption peaks of carbon on the benzene ring directly connected to the oxygen group, and 61.59 ppm is the absorption peak of methylene carbon.

### Nematocidal activity screening of title compounds

The in vitro nematocidal activity of the target compounds **5a**–**5i** was evaluated using the direct strike method against *Meloidogyne incognita*. The results showed that all of the 1,2,4-oxadiazole derivatives containing 1,3,4-thiadiazol or 1,3,4-oxadiazole moiety have low nematocidal activities. And then, introducing the trifluoromethyl pyridine moiety can enhance the activity. Of which, compounds **5n** and **5v** exhibited significant nematocidal activity against *M. incognita*, with the inhibitory ratio of 63.3% and 55.0% at 100 μg/mL, respectively, which was superior to that of tioxazafen (29.0%). There was no good activity of the 1,2,4-oxadiazole derivatives containing trifluoromethyl pyridine and diether groups (Table 1).

**Table 1:** Nematocidal and antifungal activity of compounds **5a**–**5z**

Compounds	*M. incognita*	*R. solani*
	Corrected mortality rate (%)^a、^	Inhibition rate (%)^b^
**5a**	33.4 ± 2.7	28.7 ± 1.3
**5b**	28.9 ± 5.2	15.6 ± 0.7
**5c**	37.6 ± 8.1	25.2 ± 2.2
**5d**	26.6 ± 2.0	20.4 ± 1.2
**5e**	0	18.8 ± 1.7
**5f**	29.5 ± 2.7	34.6 ± 2.3
**5** **g**	31.3 ± 5.3	16.1 ± 1.1
**5** **h**	29.4 ± 6.1	19.1 ± 0.5
**5i**	40.3 ± 1.8	23.5 ± 1.6
**5j**	39.7 ± 4.2	15.0 ± 2.3
**5** **k**	14.7 ± 27	34.7 ± 0.8
**5** **l**	31.3 ± 3.8	22.1 ± 0.8
**5** **m**	48.0 ± 3.7	23.8 ± 3.2
**5n**	63.3 ± 7.7	29.4 ± 1.0
**5o**	35.5 ± 2.4	47.0 ± 1.7
**5p**	25.3 ± 3.7	10.4 ± 0.6
**5q**	44.8 ± 6.7	26.1 ± 0.8
**5r**	36.7 ± 5.0	29.1 ± 0.5
**5** **s**	27.3 ± 8.8	13.3 ± 1.6
**5t**	35.6 ± 3.4	25.8 ± 1.3
**5u**	40.8 ± 3.4	34.5 ± 2.3
**5v**	55.0 ± 7.7	34.3 ± 0.3
**5w**	26.6 ± 4.0	32.9 ± 1.1
**5x**	47.3 ± 3.8	13.6 ± 3.2
**5y**	34.6 ± 2.4	39.4 ± 1.4
**5z**	36.5 ± 2.6	27.8 ± 1.6
Tioxazafen	29.0 ± 4.5	NT
Fluopicolide	100	NT
Azoxystrobin	NT	100

Average of three replicates

*NT* not tested

^a^ Target compounds **5a**-**5z** at a concentration of 100 μg/mL against J2 of *M. incognita*

^b^ Target compounds **5a**-**5z** at a concentration of 50 μg/mL against *R.solani*

### Antifungal activity screening of title compounds

Antifungal activity of target compounds was evaluated by using the mycelium growth method. Unfortunately, the results revealed that almost all the compounds failed to exhibit a noticeable fungicidal activity (≥ 50.0%) against *R. solani* at 50 μg/mL (Table 1).

### Antibacterial activity screening of title compounds

In vitro bacterial activity test was performed using the turbidity method and the results were listed in Table 2. As shown in Table 2, thioether derivatives containing an 1,2,4-oxadiazole scaffold have low antibacterial activities. Some target compounds introducing the trifluoromethyl pyridine moiety showed better antibacterial activities against *Xoo* and *Xoc* at a concentration of 50 μg/mL compared to the control drugs, for example compounds **5m**, **5r**, **5u**, **5v**, **5x**, and **5y** with the values of 65.85, 71.89, 64.97, 85.37, 61.97, and 78.44% against *Xoo*, respectively. Meanwhile, Compounds **5p**, **5u**, and **5v** with the values of 64.53, 65.10, and 64.59% against *Xoo*, respectively. The half maximal effective concentration (EC_50_) value of compounds was further tested. The results clearly showed that some target compounds exhibit better antibacterial activity than that of bismerthiazol (BMT) and thiodiazole copper (TDC). Compounds **5m**, **5r**, **5u**, **5v**, **5x** and **5y** showed excellent antibacterial effects on *Xoo,* with EC_50_ values of 36.25, 24.14, 28.82, 19.44, 25.37 and 28.52 μg/mL, respectively, stronger than BMT (EC_50_ = 77.46 μg/mL) and TDC (EC_50_ = 99.31 μg/mL). Compounds **5n**, **5p**, **5t**, **5u**, **5v** and **5z** exhibited strong antibacterial ability against *Xoc*, with EC_50_ values of 50.93, 31.40, 56.50, 19.04, 21.78, and 55.32 μg/mL, respectively, superior to the control agents BMT (EC_50_ = 68.50 μg/mL) and TDC (EC_50_ = 91.05 μg/mL). Among these target compounds, compound **5v** and **5u** showed the best antibacterial activity on *Xoo* and *Xoc*, respectively.

**Table 2:** Antibacterial activity of the target compounds **5a**–**5z** against *Xoo* and *Xoc*

Compounds	*Xoo*		*Xoc*	
	50 µg/mL (%)	EC_50_ (µg/mL)	50 µg/mL (%)	EC_50_ (µg/mL)
**5a**	13.50 ± 3.33	NT	17.20 ± 1.60	NT
**5b**	0	NT	0	NT
**5c**	29.00 ± 3.90	NT	20.40 ± 1.00	NT
**5d**	0	NT	0	NT
**5e**	0	NT	0	NT
**5f**	6.70 ± 4.30	NT	12.60 ± 1.10	NT
**5** **g**	0	NT	0	NT
**5** **h**	10.10 ± 3.20	NT	11.30 ± 5.30	NT
**5i**	10.90 ± 1.00	NT	19.40 ± 2.10	NT
**5j**	42.17 ± 1.39	74.70 ± 5.46	41.37 ± 5.42	88.47 ± 4.21
**5** **k**	30.59 ± 3.19	85.30 ± 3.66	15.58 ± 2.84	199.65 ± 18.98
**5** **l**	14.16 ± 2.93	129.15 ± 11.16	29.95 ± 3.57	172.18 ± 13.94
**5** **m**	65.85 ± 3.32	36.25 ± 1.77	33.01 ± 3.23	76.35 ± 4.82
**5n**	42.77 ± 2.13	56.75 ± 5.98	53.73 ± 3.24	50.93 ± 8.07
**5o**	26.94 ± 0.65	122.80 ± 7.32	31.60 ± 5.28	108.27 ± 5.99
**5p**	11.99 ± 1.18	225.35 ± 8.03	64.53 ± 3.42	31.40 ± 2.07
**5q**	43.78 ± 2.02	63.32 ± 1.58	33.61 ± 4.13	123.34 ± 8.55
**5r**	71.89 ± 1.56	24.14 ± 2.72	48.53 ± 1.66	51.11 ± 3.05
**5** **s**	26.30 ± 2.37	95.12 ± 14.87	30.28 ± 2.88	116.76 ± 13.97
**5t**	38.16 ± 2.27	102.45 ± 5.99	50.37 ± 3.23	56.50 ± 6.26
**5u**	64.97 ± 3.46	28.82 ± 2.25	65.10 ± 4.12	19.04 ± 2.13
**5v**	85.37 ± 1.46	19.44 ± 2.22	64.59 ± 4.43	21.78 ± 2.82
**5w**	40.60 ± 3.95	75.61 ± 2.21	40.12 ± 3.23	75.22 ± 3.93
**5x**	61.97 ± 0.92	25.37 ± 6.98	40.29 ± 3.84	101.96 ± 5.81
**5y**	78.44 ± 5.54	28.52 ± 3.14	20.63 ± 6.75	125.57 ± 15.77
**5z**	44.07 ± 2.36	66.51 ± 5.53	53.70 ± 3.73	55.32 ± 2.96
BMT	35.12 ± 2.72	77.46 ± 6.15	45.56 ± 5.76	68.50 ± 7.87
TDC	25.12 ± 3.31	99.31 ± 7.59	37.58 ± 2.84	91.05 ± 8.84

Average of three replicates

*NT* not tested

**Fig. 2:**
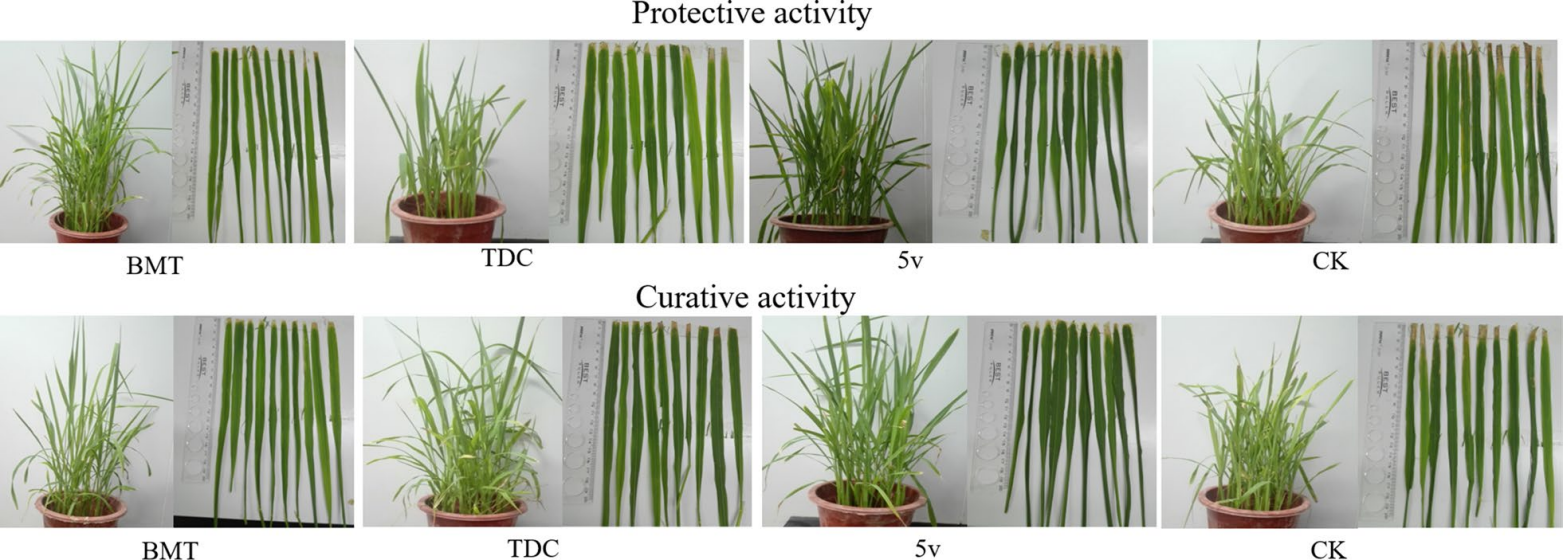
Protective activity and curative of compound **5v** against rice leaf blight under greenhouse conditions at a concentration of 200 μg/mL

Compound **5v** exhibited excellent antibacterial ability to *Xoo*, with an EC_50_ value of 19.44 μg/mL. Accordingly, the control effect of compound **5v** on rice bacterial leaf blight was evaluated and the results were showed in Table 3 and Fig. 2. Compound **5v** exerted moderate control effects on rice bacterial leaf blight at 200 μg/mL, with curative and protective activity values of 37.8% and 27.6%, respectively, lower than those of the control agents BMT (46.7% and 36.1%, respectively) and TDC (31.8% and 30.9%, respectively).

**Table 3:** Curative and protective activity of compound **5v** against rice bacterial leaf blight

Treatments	Protective activity		Curative activity	
	Disease index (%)	Control effect (%)^a^	Disease index (%)	Control effect (%)^a^
**5v**	60.6	27.6 ± 3.7	50.1	37.8 ± 4.2
BMT	54.2	36.1 ± 2.5	43.3	46.7 ± 1.8
TDC	56.5	30.9 ± 3.4	56.2	31.8 ± 5.6
Negative control	84.3	–	81.2	–

Compounds **5v** at a concentration of 200 μg/mL against rice bacterial leaf blight

^a^Statistical analysis was conducted using the analysis of variance method

The preliminary structure–activity relationship analysis results were as follows: Firstly, the introduction of strong electron withdrawing groups, such as –NO_2_ (**5n**, EC_50_ = 50.93 μg/mL) and 4-CF_3_ (**5o**, EC_50_ = 108.27 μg/mL), into the benzene ring group of mono-ether structure compounds **5j–5r**, culminated in either a significant or non-significant improvement in antibacterial activity; alkyl groups did not evoke a significant improvement over phenyl. Secondly, compared to other substituents, we found that the introduction of fluorine or chlorine (compounds **5u** and **5v**) at the 4-position of the phenyl group exerted the most significant effect on improving antibacterial activity against *Xoo* and *Xoc*; confusingly, continue compounds **5v** to introduce chlorine atom (such as compound 5 s with 2,4-Cl, EC_50_ = 116.76 μg/mL) on the benzene ring second position was overall antibacterial ability has not improved but has declined. Finally, introduction of electron withdrawing group or electron donating group of thioether structure compounds **5a**–**5i** disfavor to their activities.

## Conclusions

In summary, twenty-six novel 1,2,4-oxadiazole derivatives containing 1,3,4-thiadiazol (1,3,4-oxadiazole) and trifluoromethyl pyridine moieties were synthesized based on 1,2,4-oxadiazole pharmacophore. Compound **5n** and **5v** exhibited excellent nematocidal activity, superior to leading compound Tioxazafen. What’s more notable is that compound **5v** and **5u** showed significantly inhibitory activity against the plant pathogenic bacteria *Xoo* and *Xoc*, respectively, higher than commercial bactericide BMT and TDC. The present study demonstrated the potential of 1,2,4-oxadiazole ether derivatives as effective nematocidal and antimicrobial agents for crop protection and should serve as a basis for future studies.

## Supplementary information

**Additional file 1.** The ^1^H NMR, ^13^C NMR, and HRMS data of target compounds.

## Data Availability

All data generated or analysed during this study are included in this published article [and its Additional files].

## Abbreviations

*Xoo*: *Xanthomonas oryzae* pv. *oryzae*
*Xoc*: *Xanthomonas oryzae* pv. *oryzicola*
BMT: Bismerthiazol
TDC: Thiodiazole copper
^1^H NMR: ^1^H nuclear magnetic resonance
^13^C NMR: ^13^C nuclear magnetic resonance
HRMS: High resolution mass spectrum
