# Supplementary material for: Design, synthesis, and biological activity of novel 1,2,4-oxadiazole derivatives
Source: BMC Chem. 2020 Nov 22;14(1):68. doi: 10.1186/s13065-020-00722-1 (PMC7680602; doi:10.1186/s13065-020-00722-1)
Supplement: Supplementary file 1 — Additional file 1. The 1H NMR, 13C NMR, and HRMS data of target compounds. [file 13065_2020_722_MOESM1_ESM.doc]

**Design, synthesis, and biological activity of novel 1,2,4-oxadiazole derivatives**

Lingzhi Zhu, Huanan Zeng, Dan Liu, Yun Fu, Qiong Wu, Baoan Song*, Xiuhai Gan*

State Key Laboratory Breeding Base of Green Pesticide and Agricultural Bioengineering, Key Laboratory of Green Pesticide and Agricultural Bioengineering, Ministry of Education, Guizhou University, Guiyang 550025, China

**The copies of ^1^H NMR, ^13^C NMR, and HRMS for the title compounds** **(5a−5z)**

**
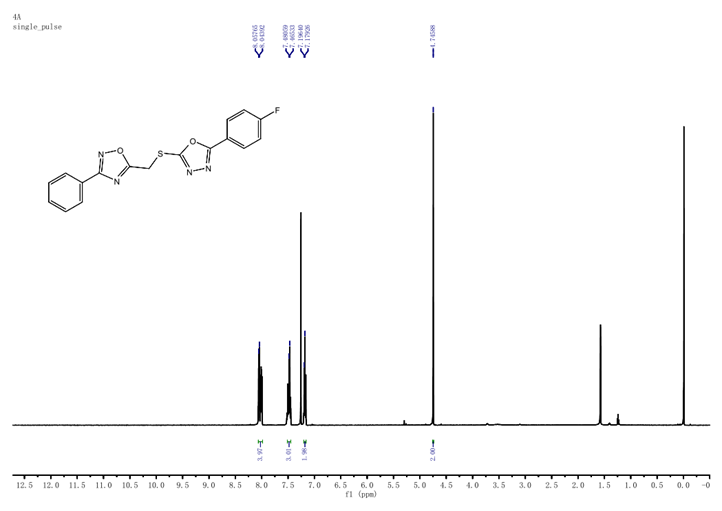
**

**Fig** **S1** ^1^H NMR of compound **5a**


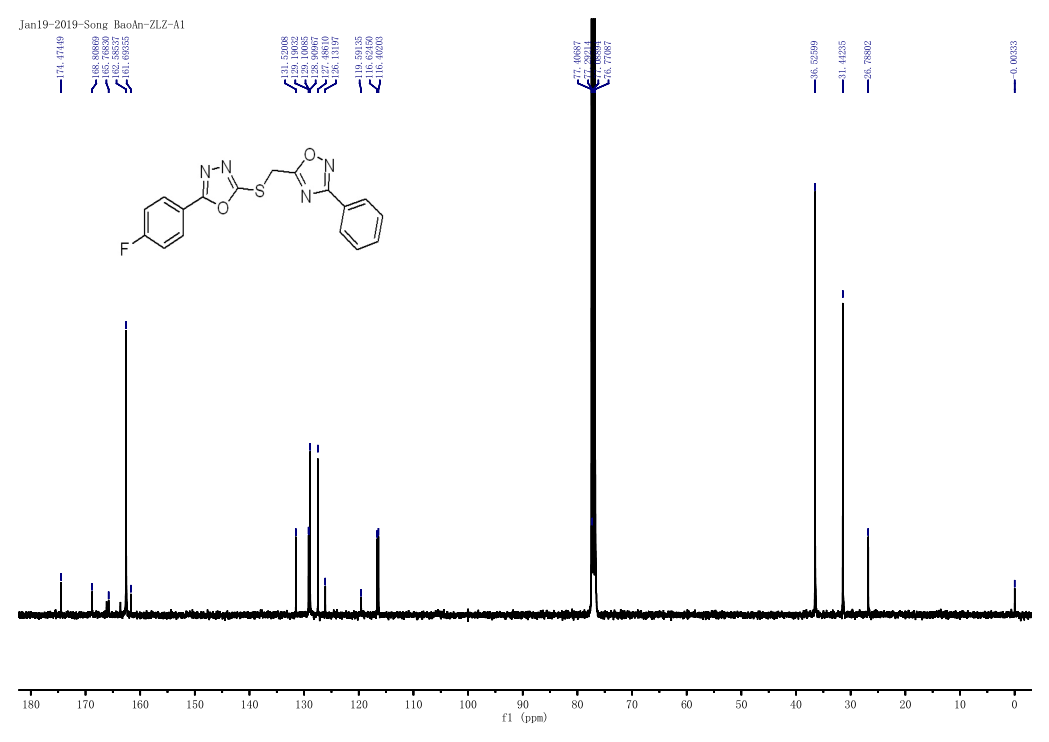


**Fig S2** ^13^C NMR of compound **5a**

**
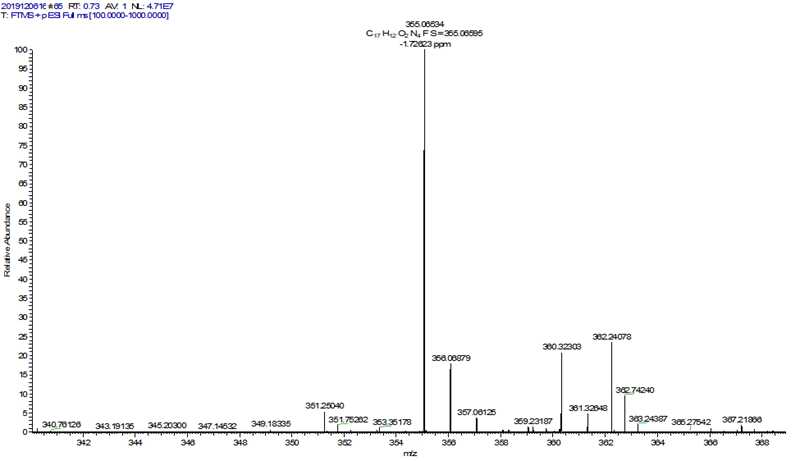
**

**Fig** **S3** HRMS of compound **5a**

**
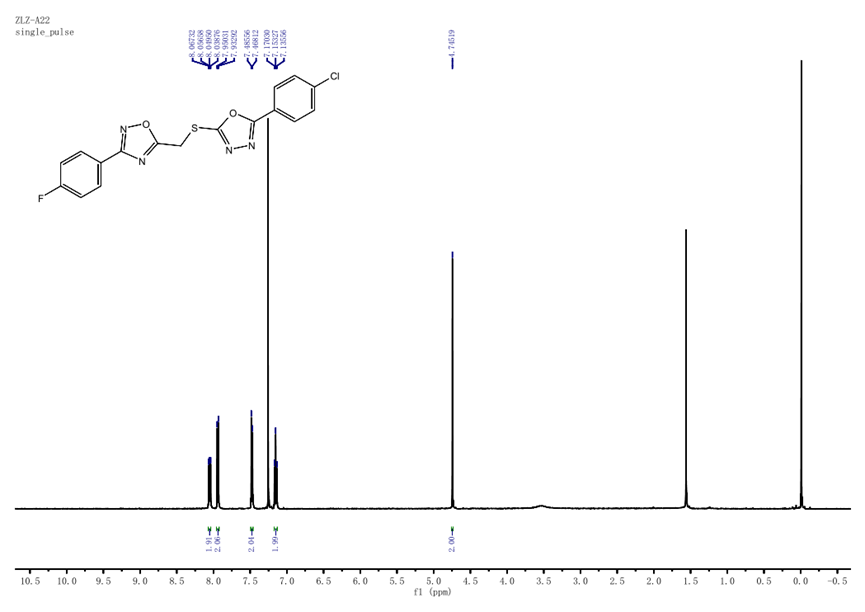
**

**Fig** **S4** ^1^H NMR of compound **5b**


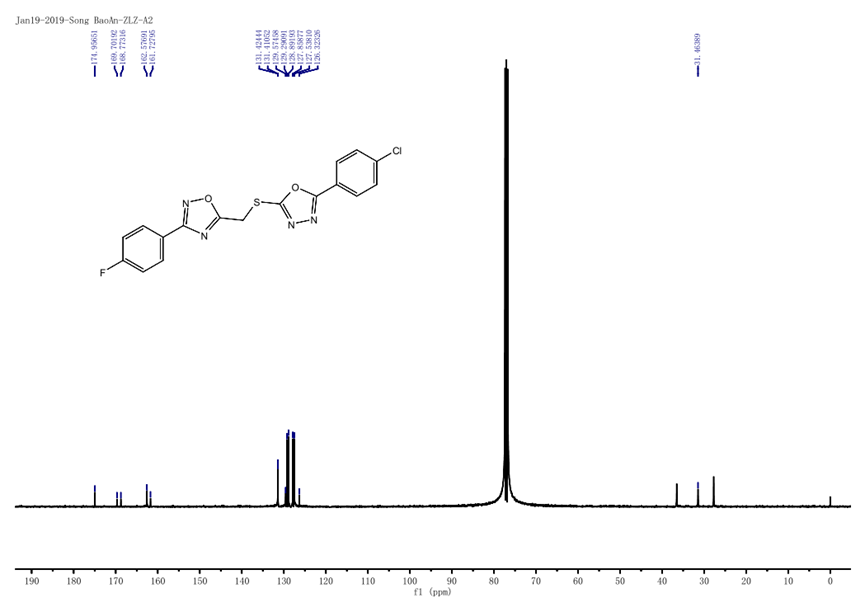


**Fig S5** ^13^C NMR of compound **5b**

**
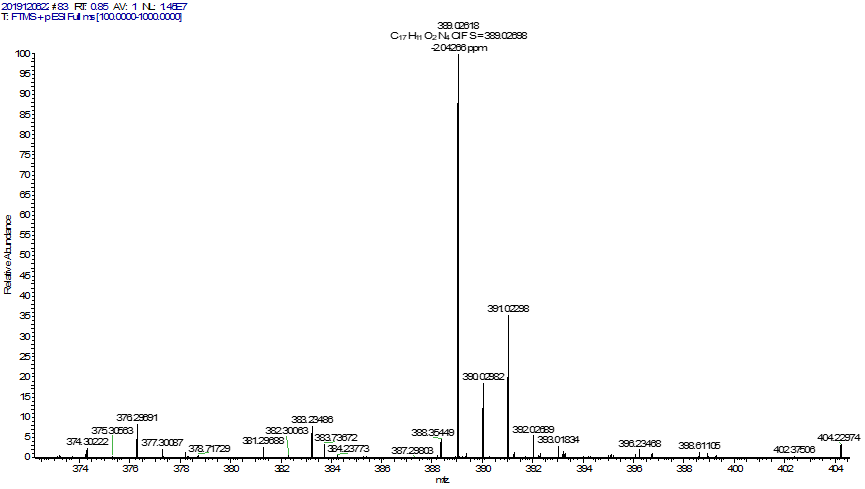
**

**Fig S6** HRMS of compound **5b**


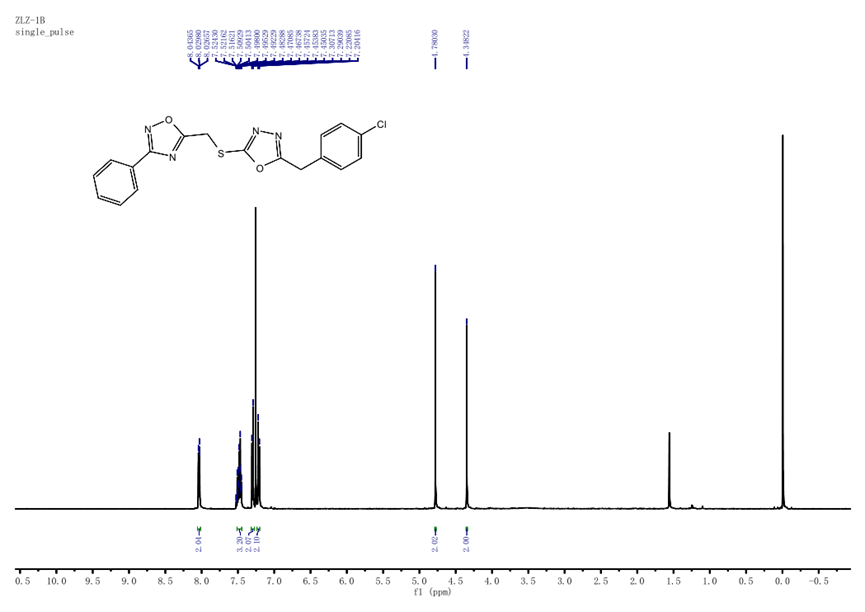


**Fig** **S7** ^1^H NMR of compound **5c**


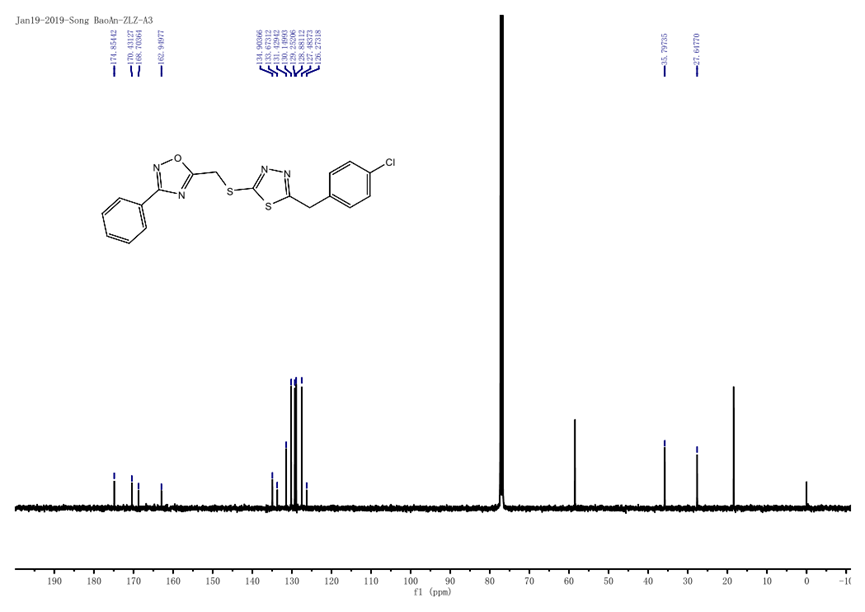


**Fig S8** ^13^C NMR of compound **5c**


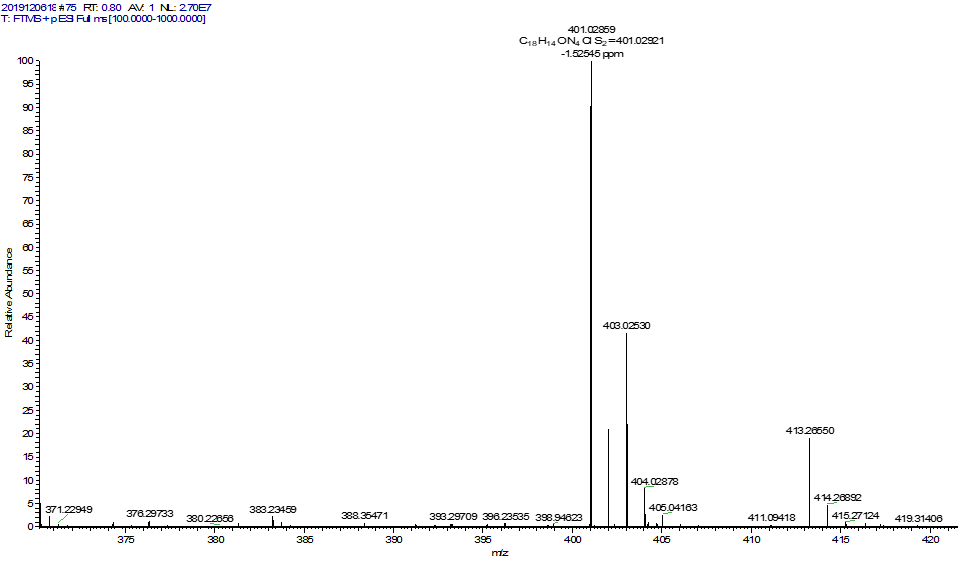


**Fig S9** HRMS of compound **5c**


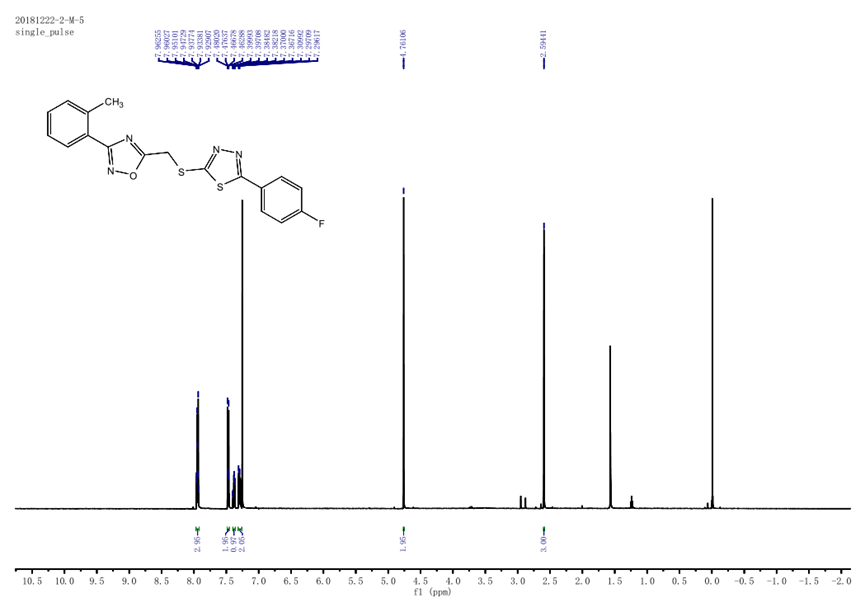


**Fig** **S10** ^1^H NMR of compound **5d**

**
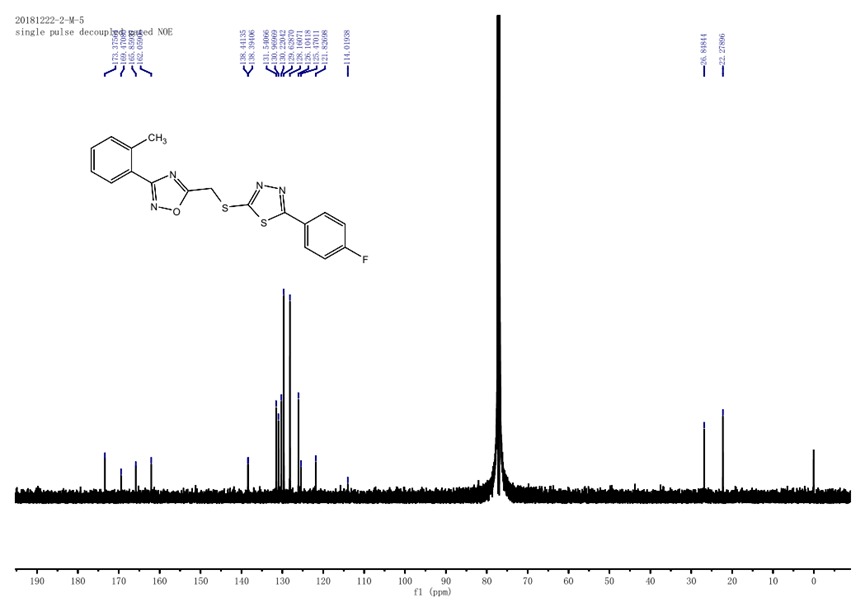
**

**Fig S11** ^13^C NMR of compound **5d**


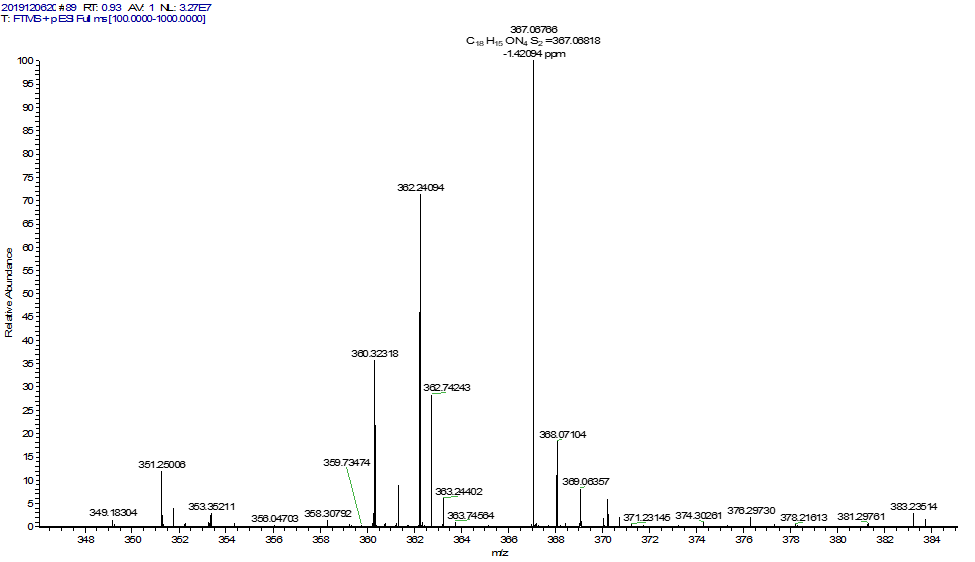


**Fig S12** HRMS of compound **5d**


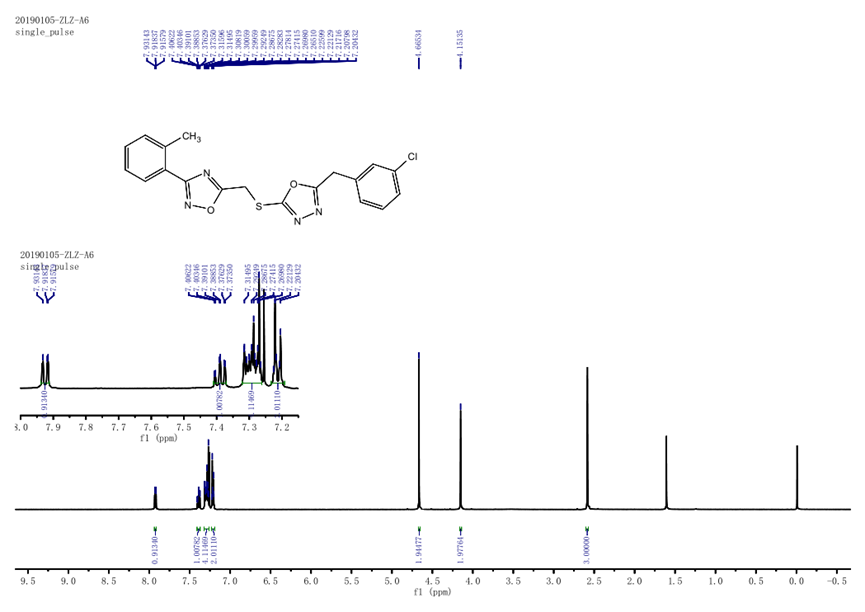


**Fig** **S13** ^1^H NMR of compound **5e**

**
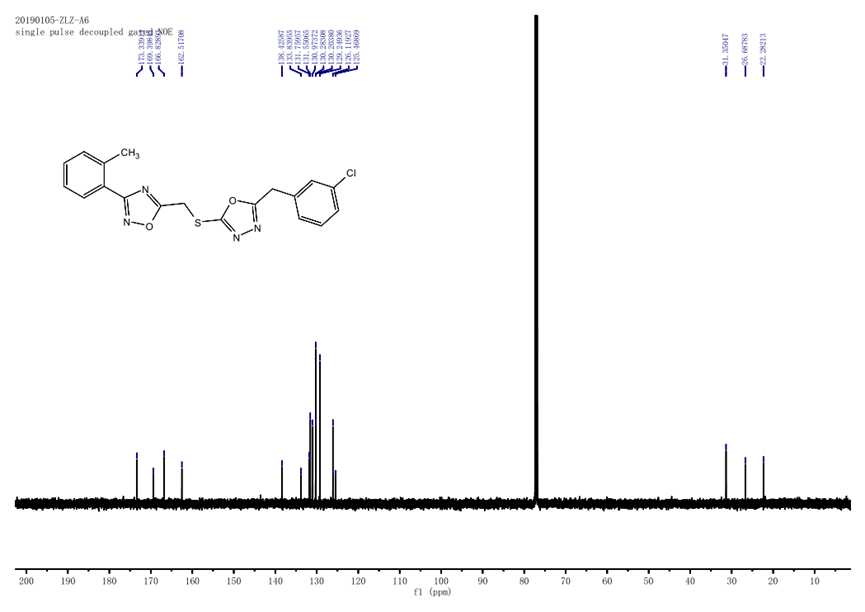
**

**Fig S14** ^13^C NMR of compound **5e**


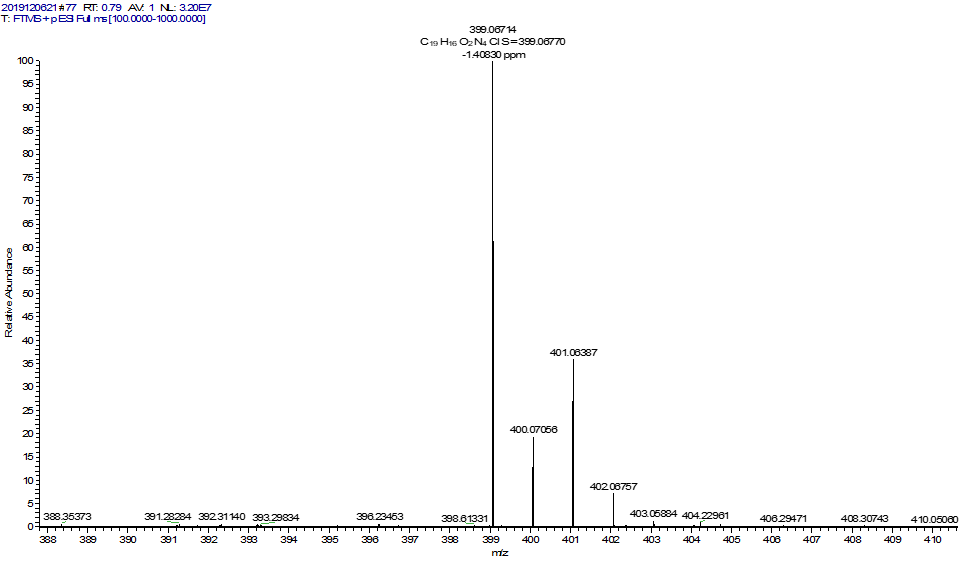


**Fig S15** HRMS of compound **5e**


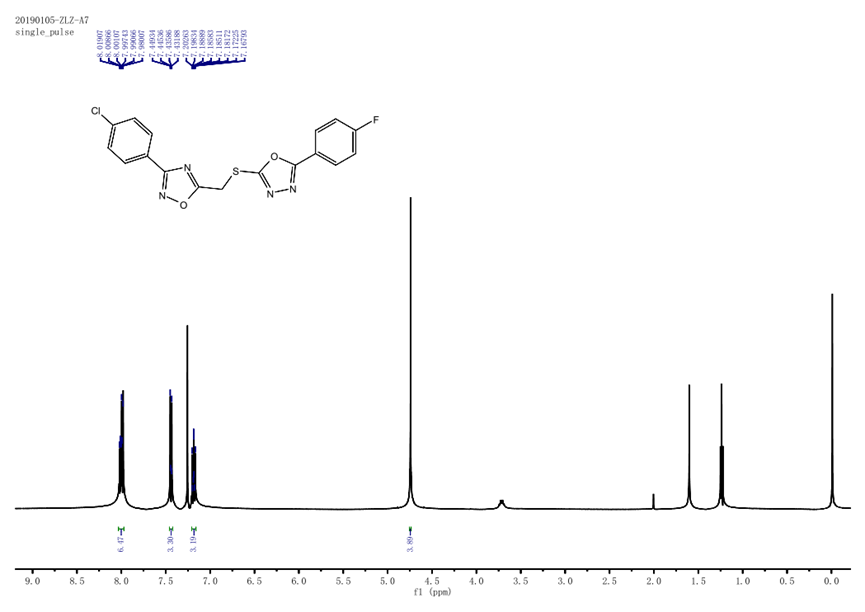


**Fig** **S16** ^1^H NMR of compound **5f**

**
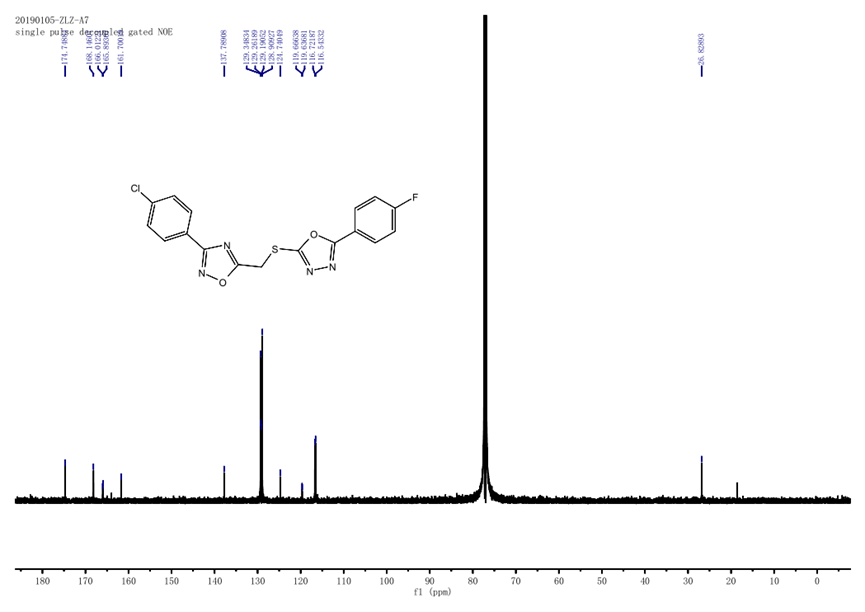
**

**Fig S17** ^13^C NMR of compound **5f**


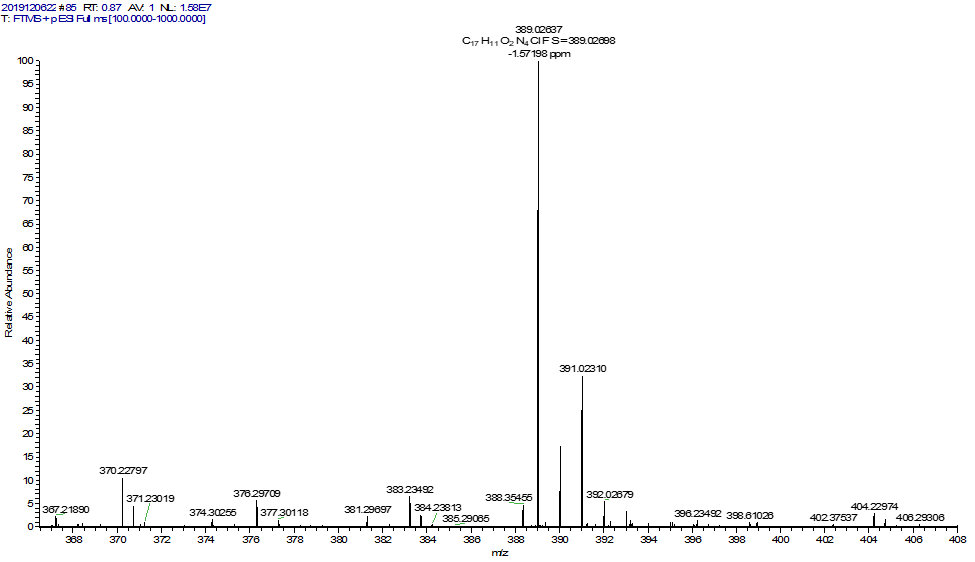


**Fig S18** HRMS of compound **5f**


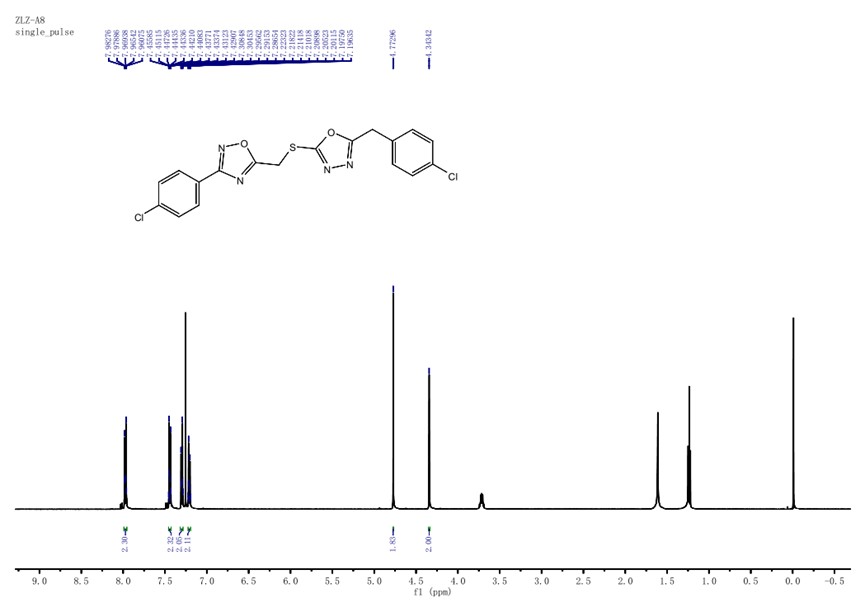


**Fig** **S19** ^1^H NMR of compound **5g**

**
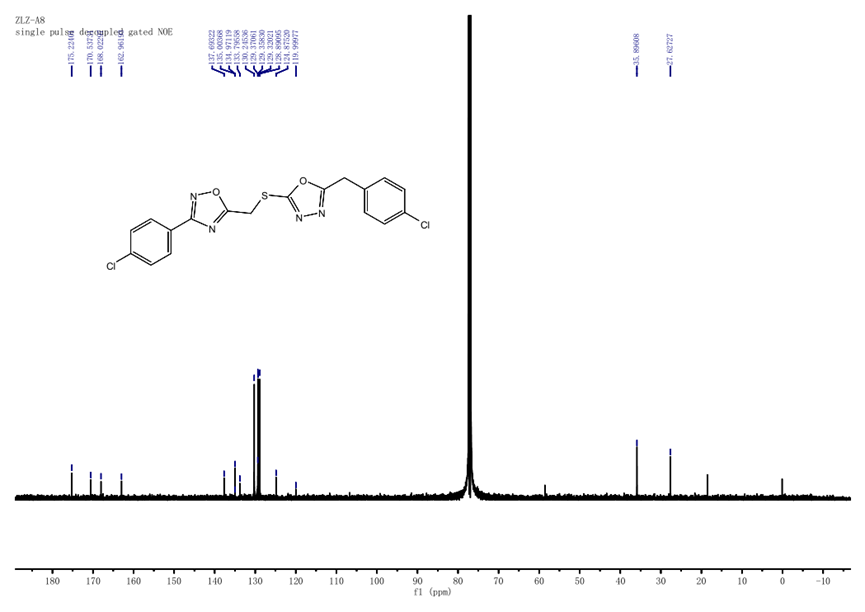
**

**Fig S20** ^13^C NMR of compound **5g**


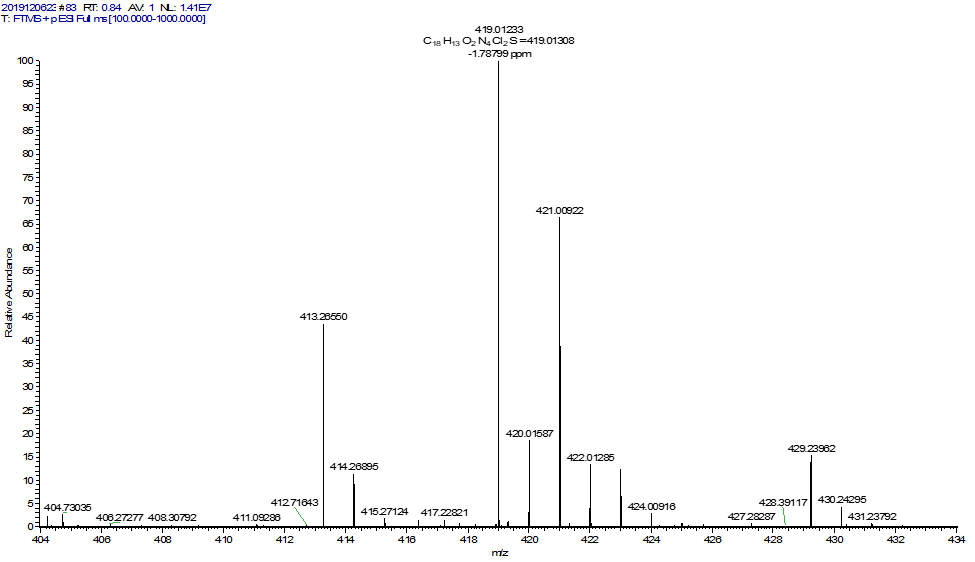


**Fig S21** HRMS of compound **5g**


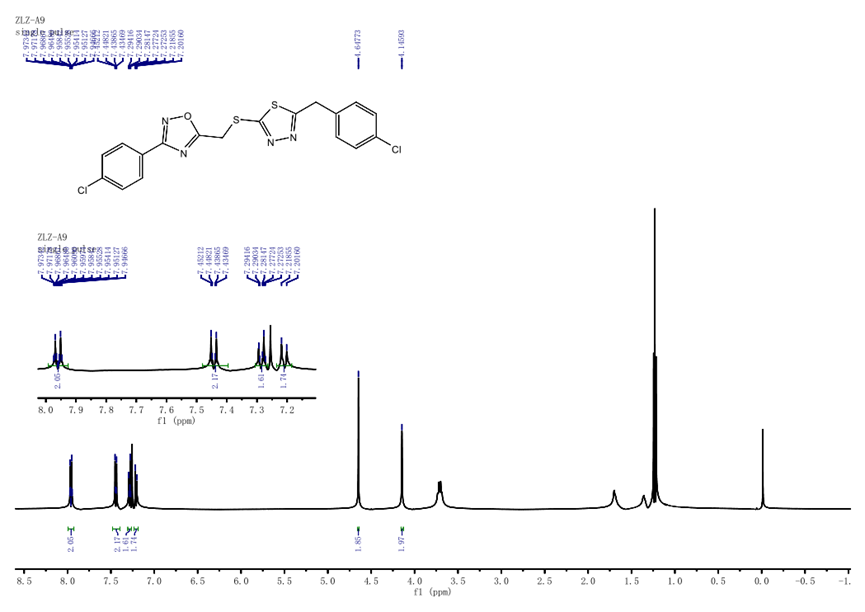


**Fig** **S22** ^1^H NMR of compound **5h**


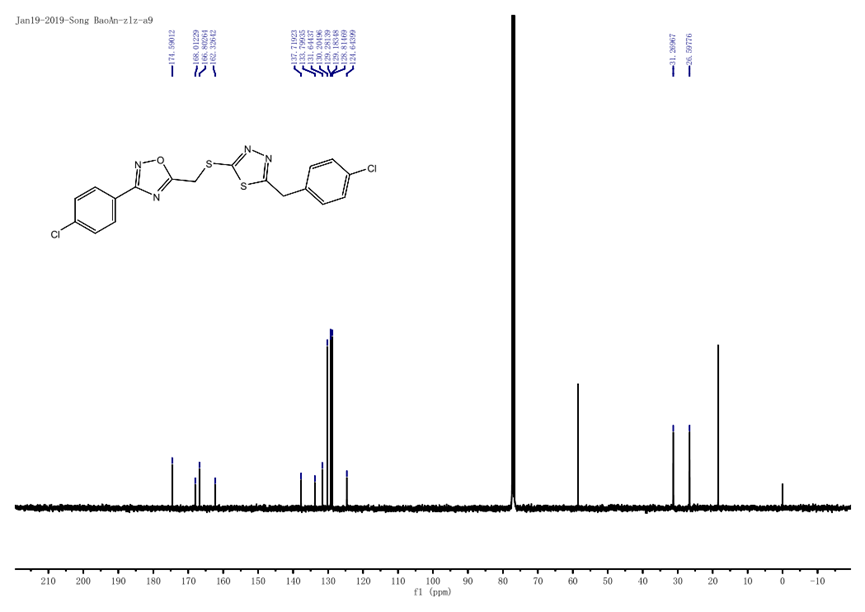


**Fig S23** ^13^C NMR of compound **5h**


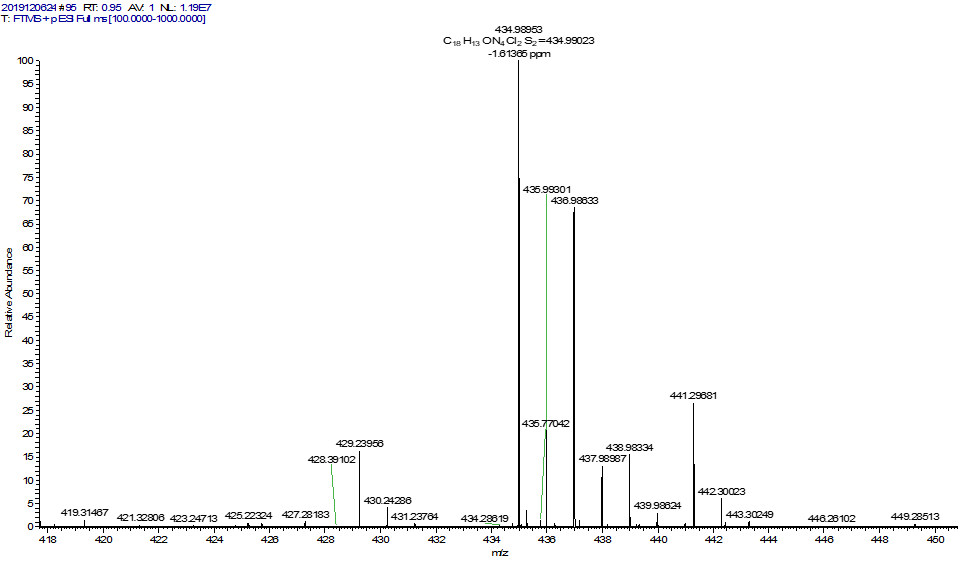


**Fig S24** HRMS of compound **5h**

**
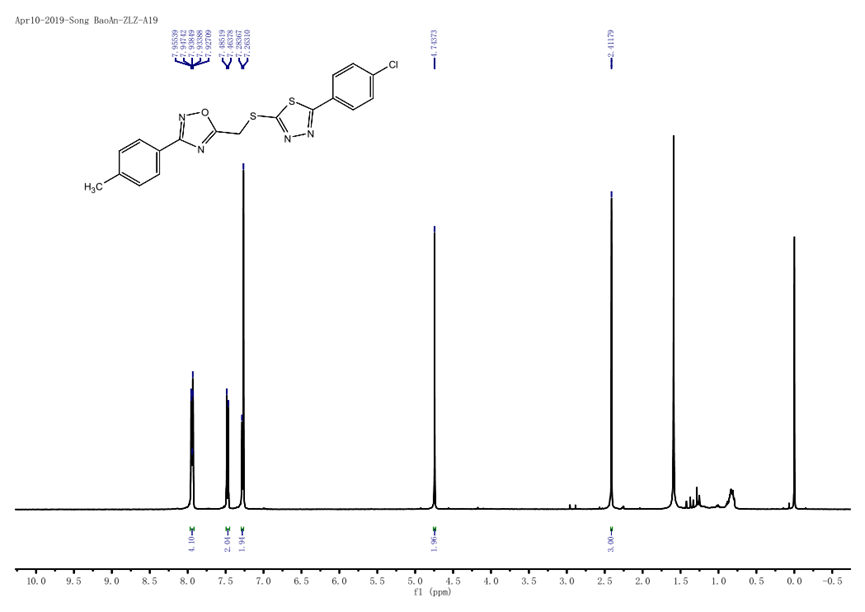
**

**Fig S25** ^1^H NMR of compound **5i**

**
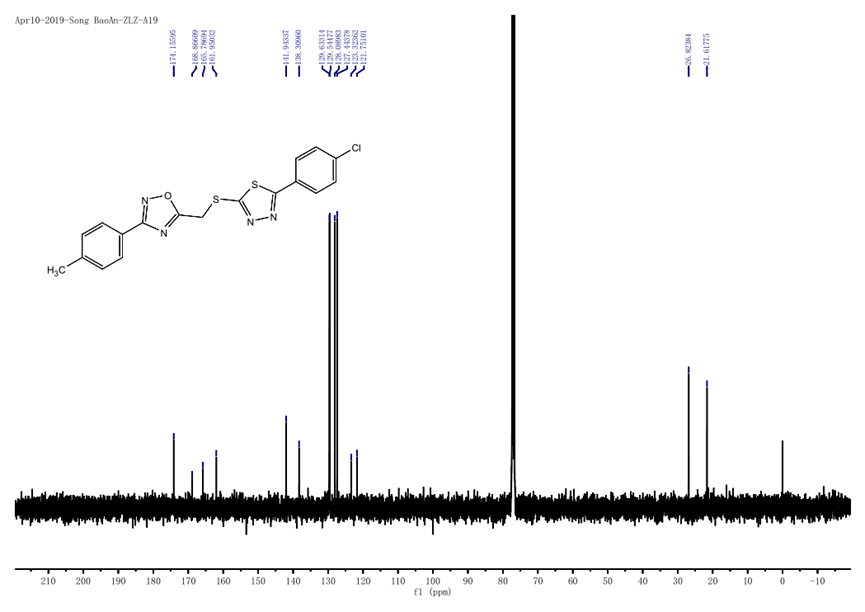
**

**Fig S26** ^13^C NMR of compound **5i**


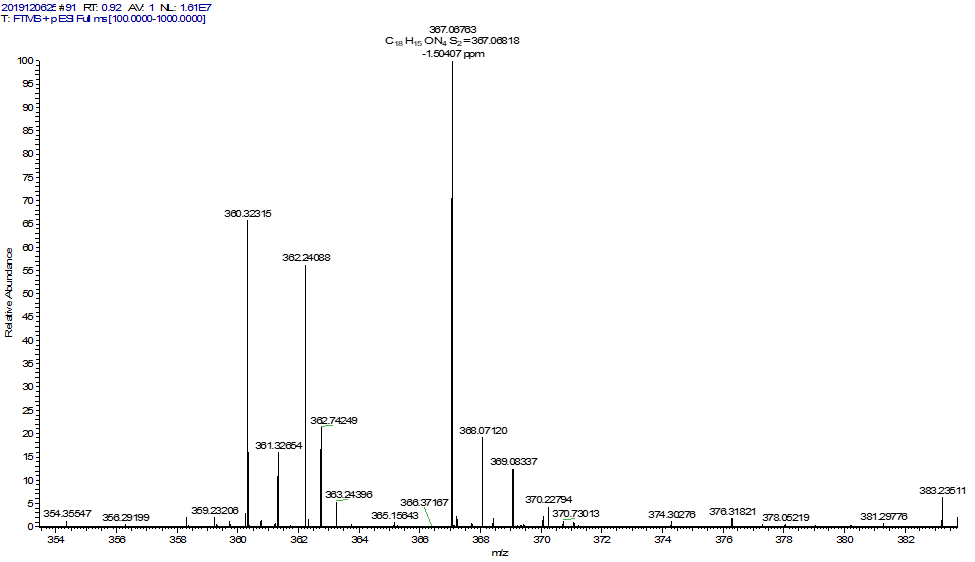


**Fig S27** HRMS of compound **5i**


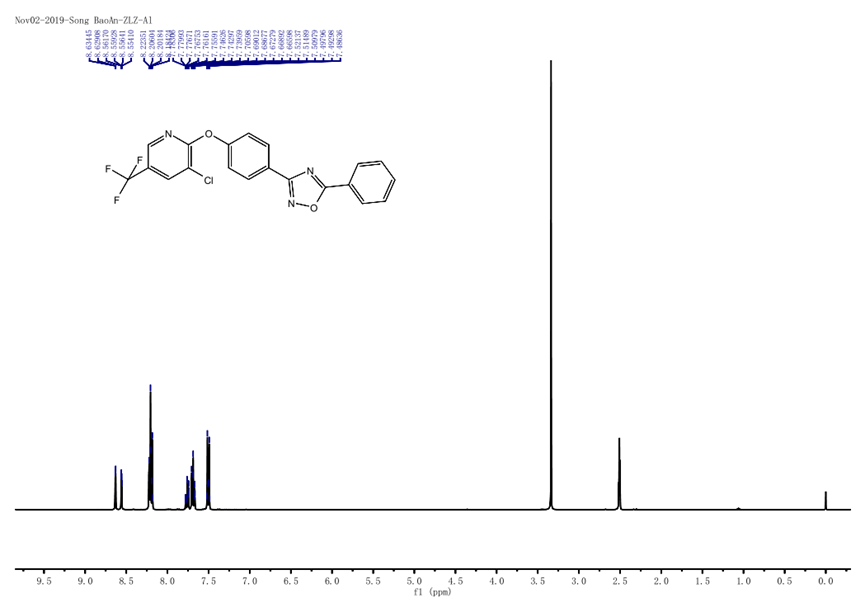


**Fig S28** ^1^H NMR of compound **5j**


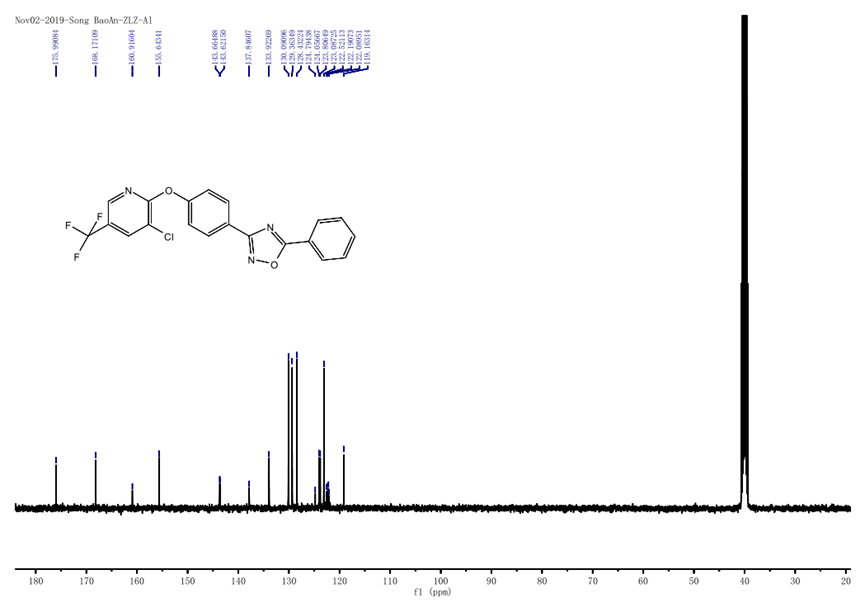


**Fig S29** ^13^C NMR of compound **5j**

**
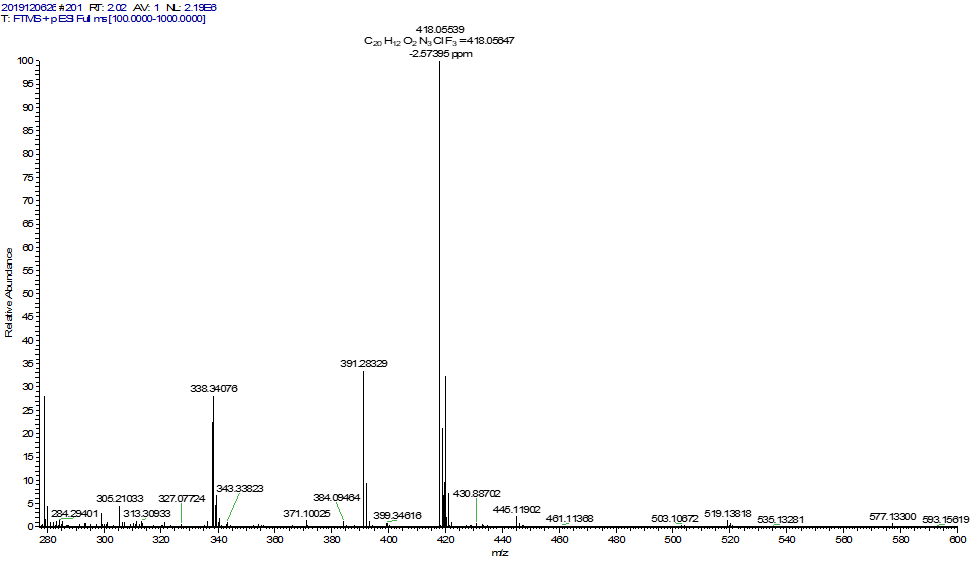
**

**Fig S30** HRMS of compound **5j**

**
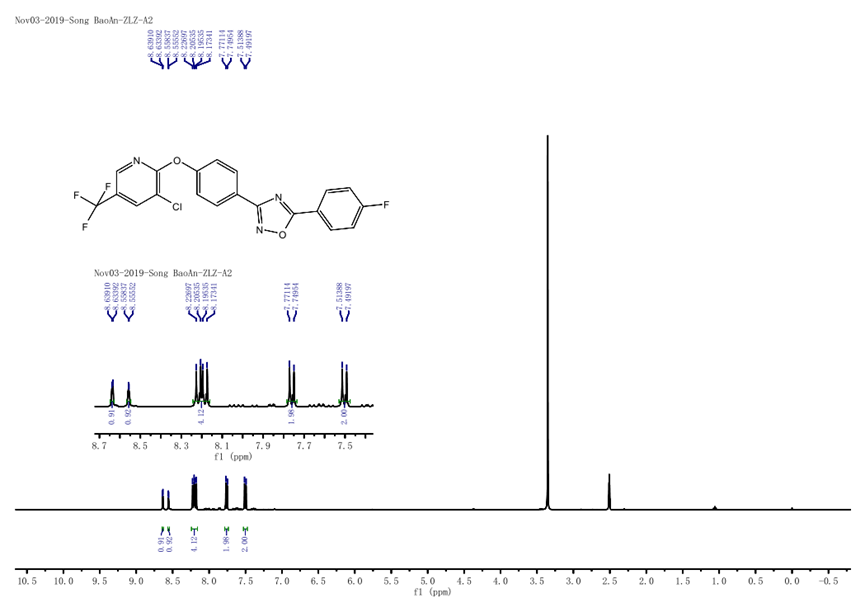
**

**Fig S31** ^1^H NMR of compound **5k**

**
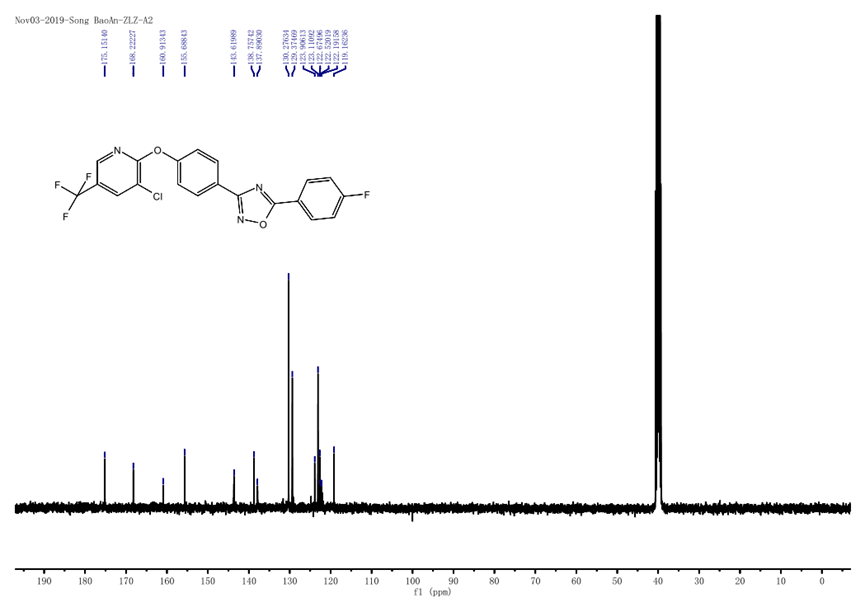
**

**Fig S32** ^13^C NMR of compound **5k**

**
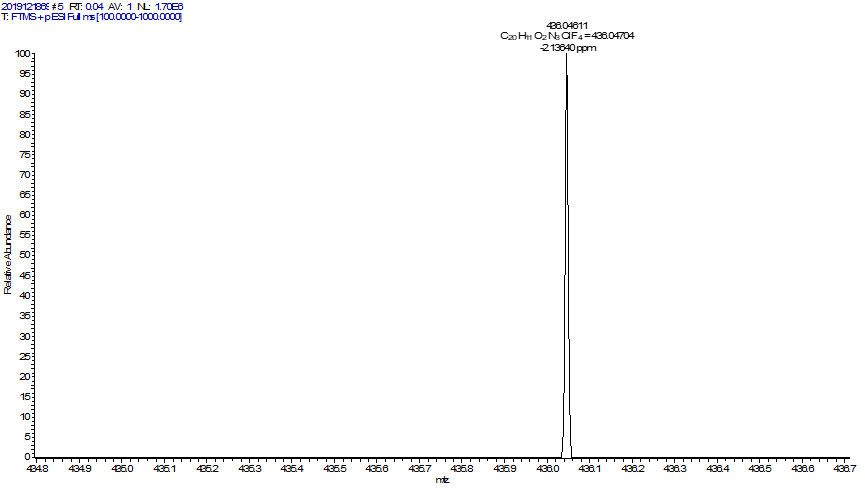
**

**Fig S33** HRMS of compound **5k**

**
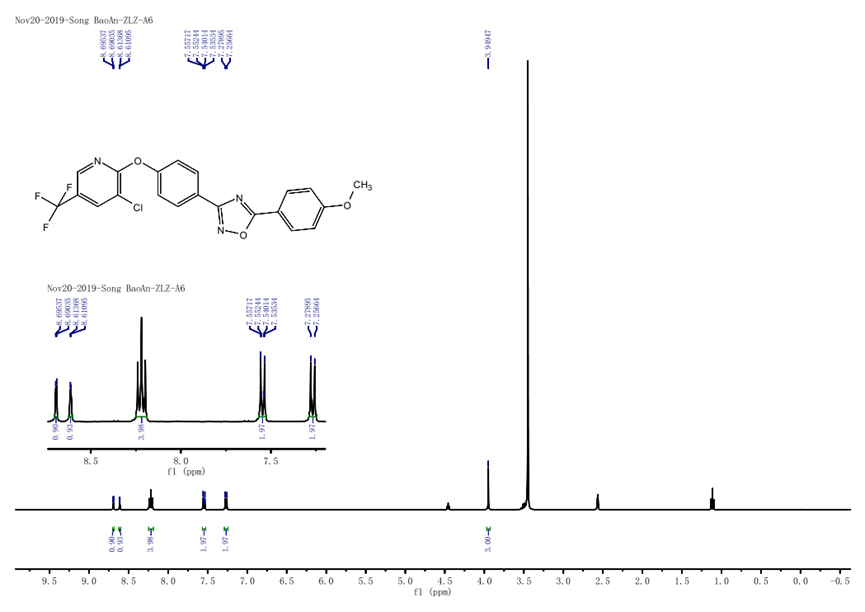
**

**Fig S34** ^1^H NMR of compound **5l**

**
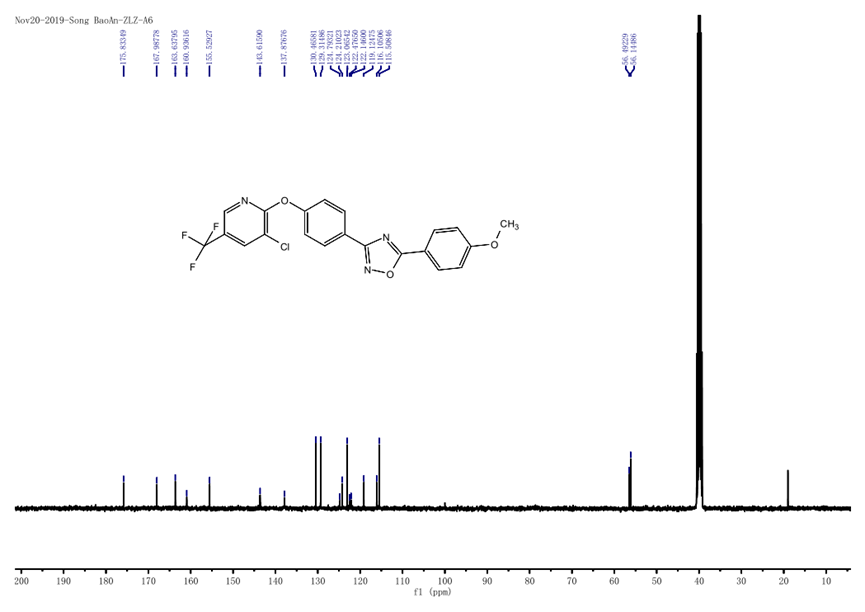
**

**Fig S35** ^13^C NMR of compound **5l**

**
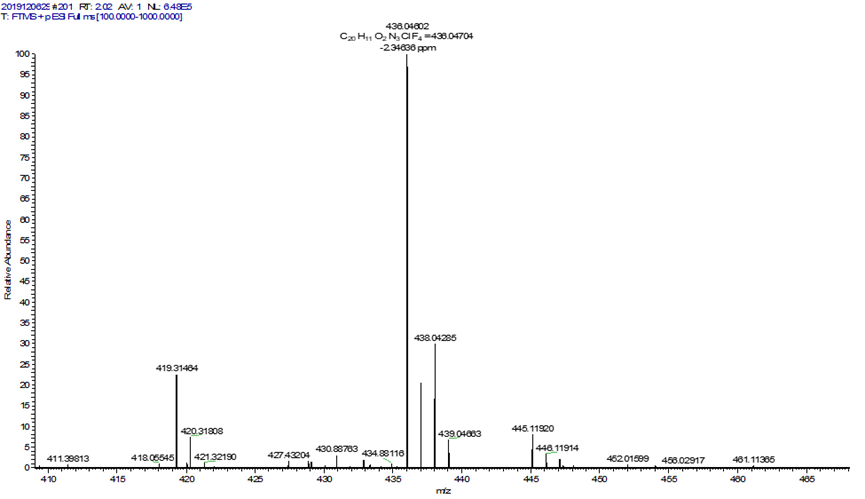
**

**Fig S36** HRMS of compound **5l**


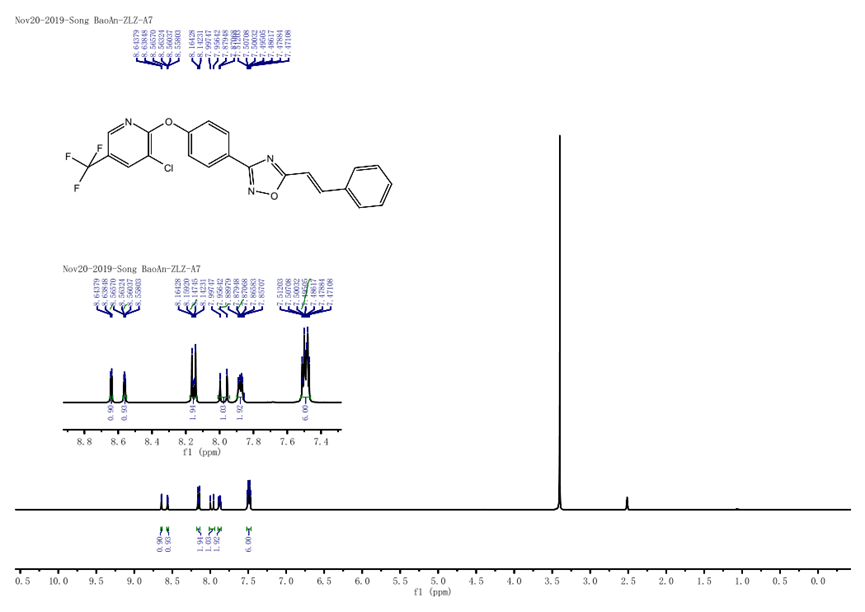


**Fig S37** ^1^H NMR of compound **5m**


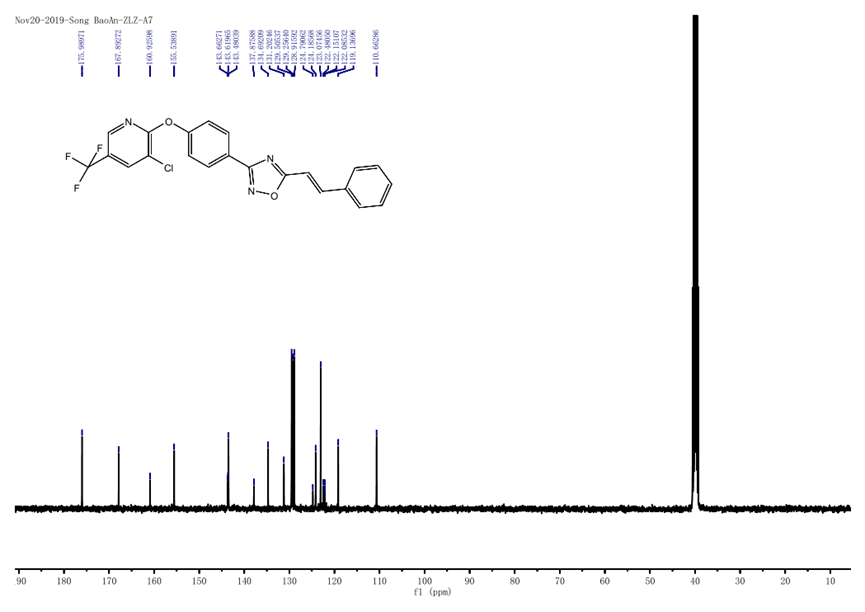


**Fig S38** ^13^C NMR of compound **5m**


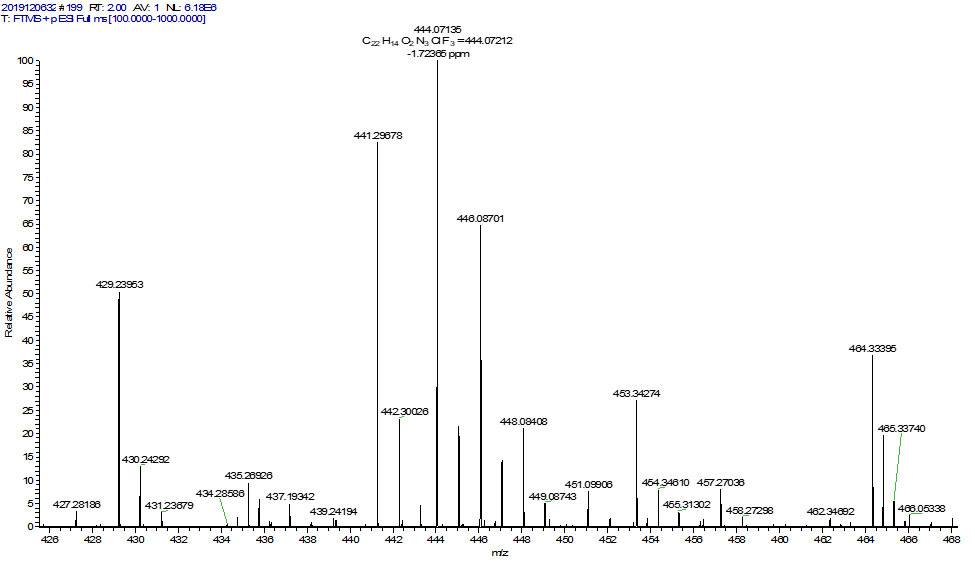


**Fig S39** HRMS of compound **5m**

**
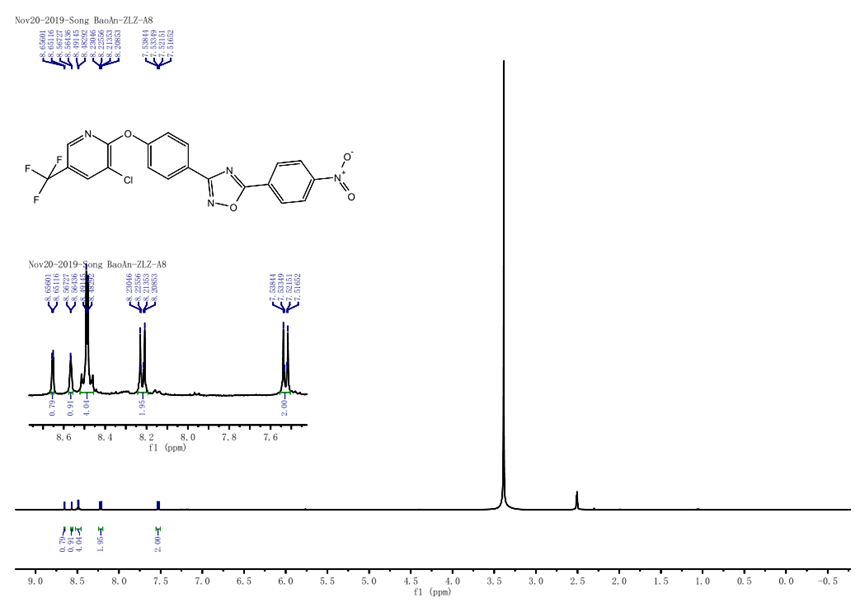
**

**Fig S40** ^1^H NMR of compound **5n**

**
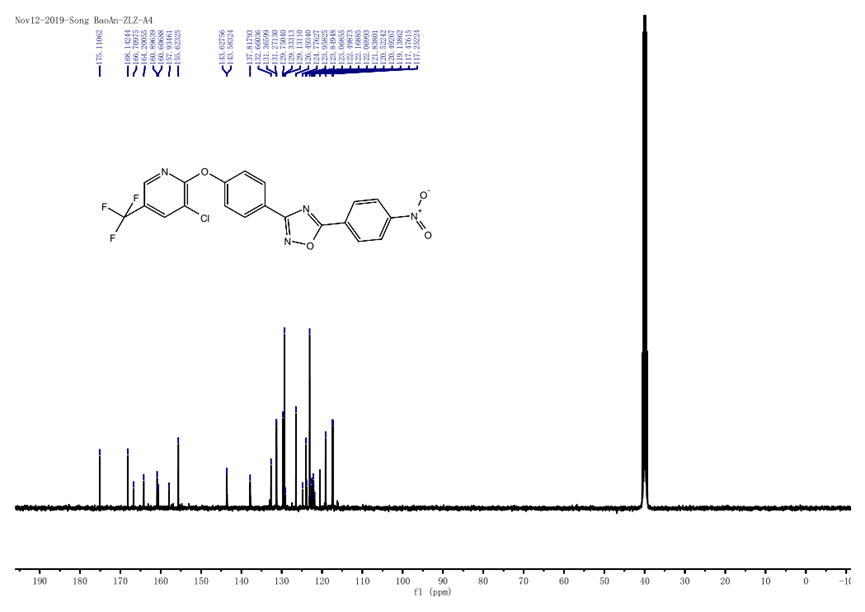
**

**Fig S41** ^13^C NMR of compound **5n**

**
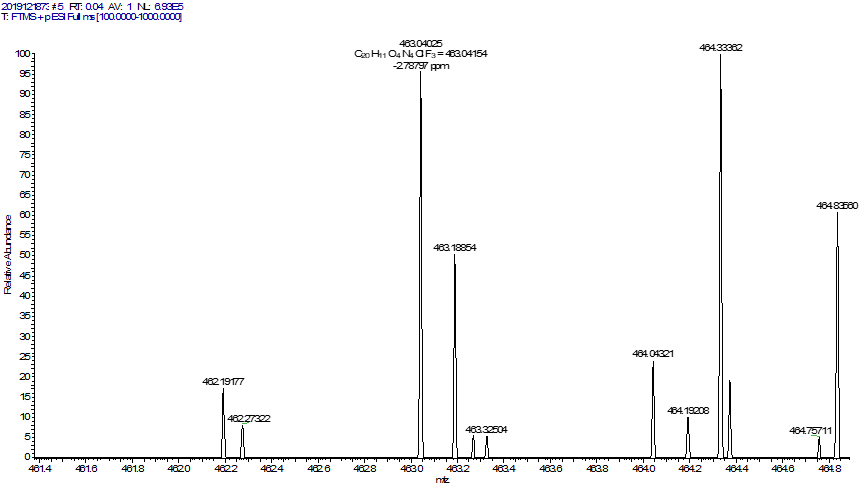
**

**Fig S42** HRMS of compound **5n**

**
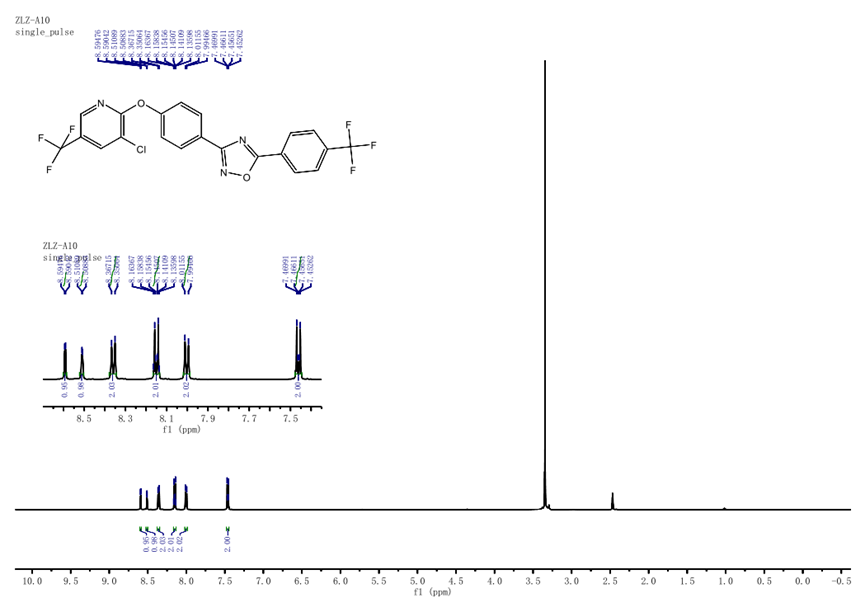
**

**Fig S43** ^1^H NMR of compound **5o**

**
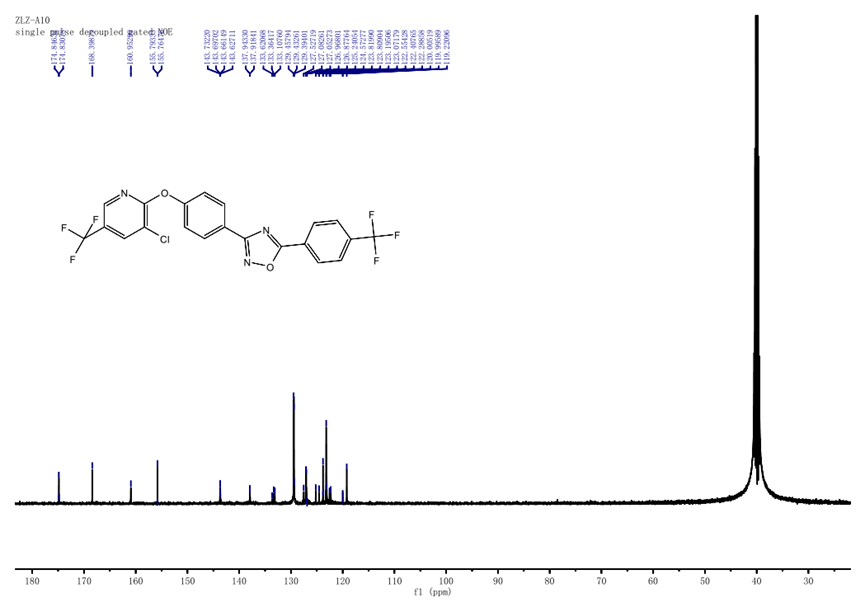
**

**Fig S44** ^13^C NMR of compound **5o**

**
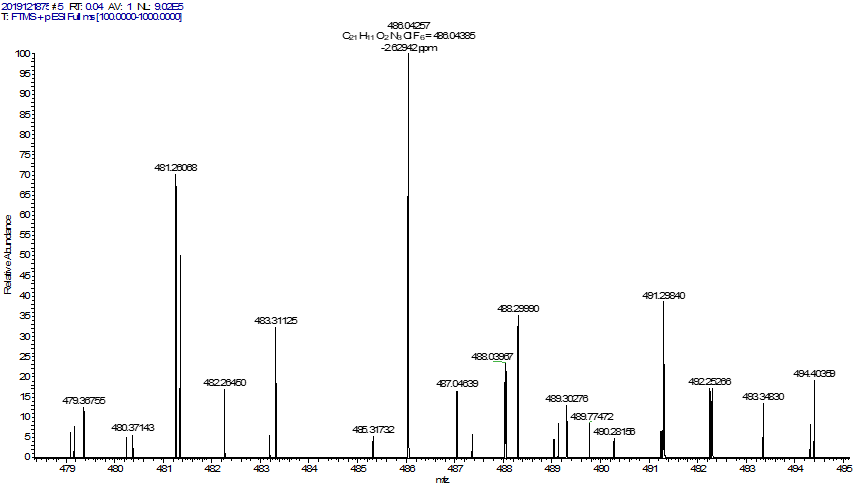
**

**Fig S45** HRMS of compound **5o**

**
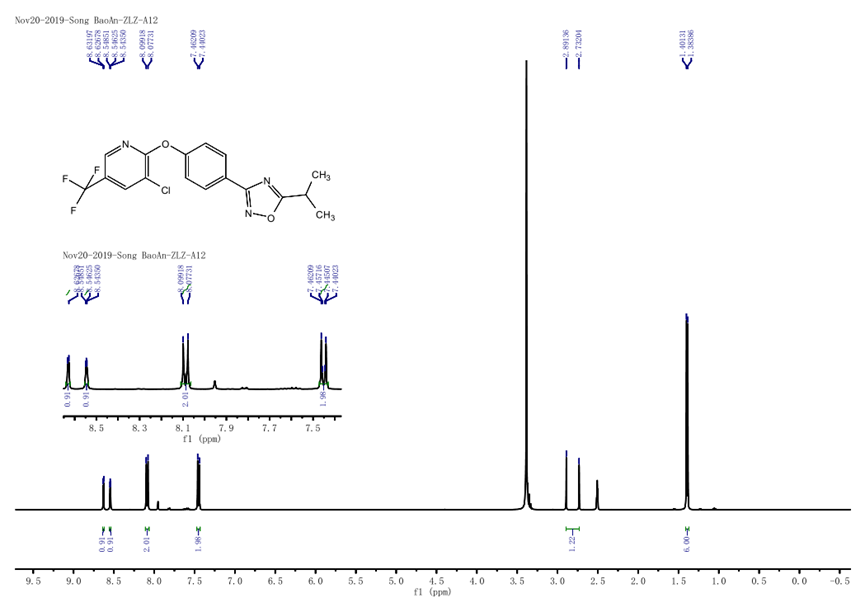
**

**Fig S46** ^1^H NMR of compound **5p**

**
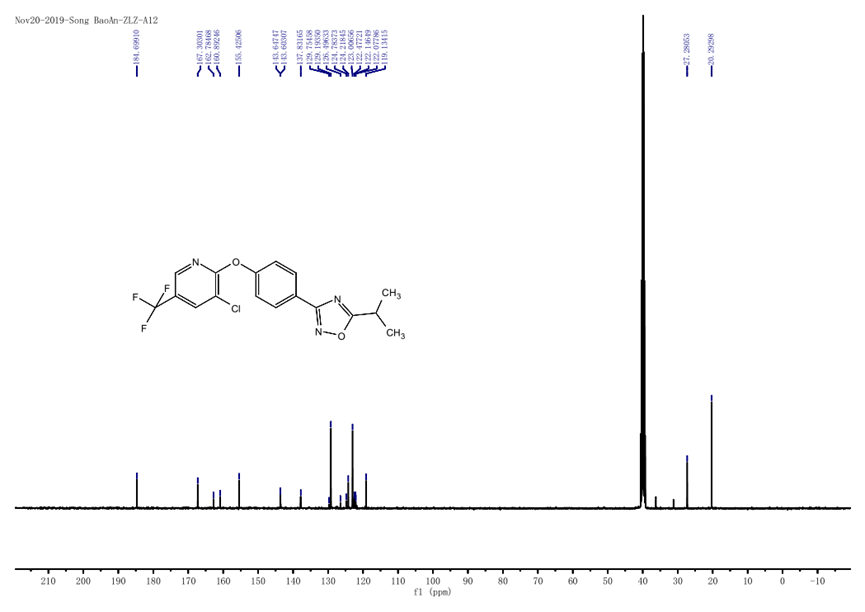
**

**Fig S47** ^13^C NMR of compound **5p**


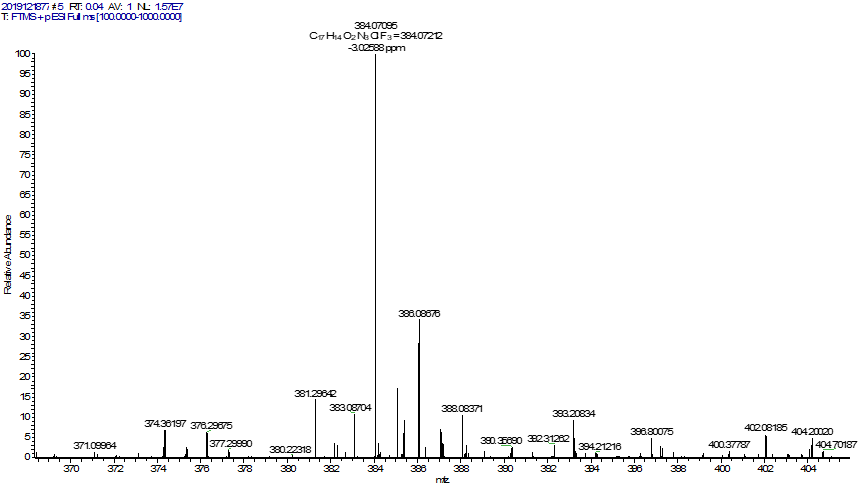


**Fig S48** HRMS of compound **5p**

**
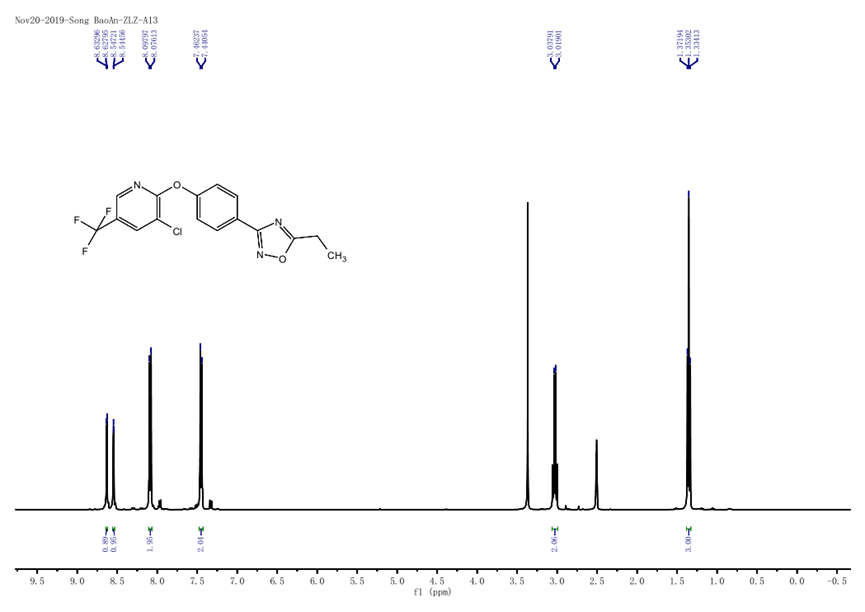
**

**Fig S49** ^1^H NMR of compound **5q**

**
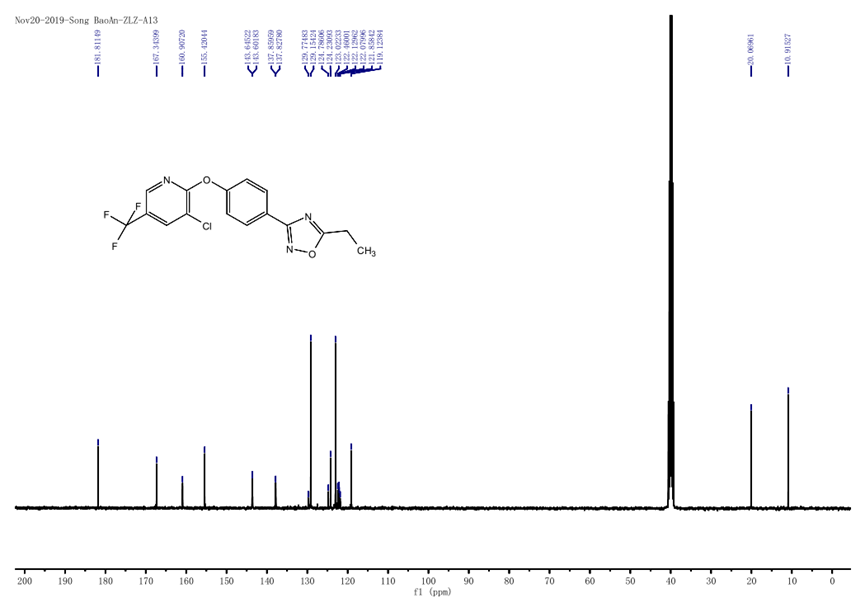
**

**Fig S50** ^13^C NMR of compound **5q**


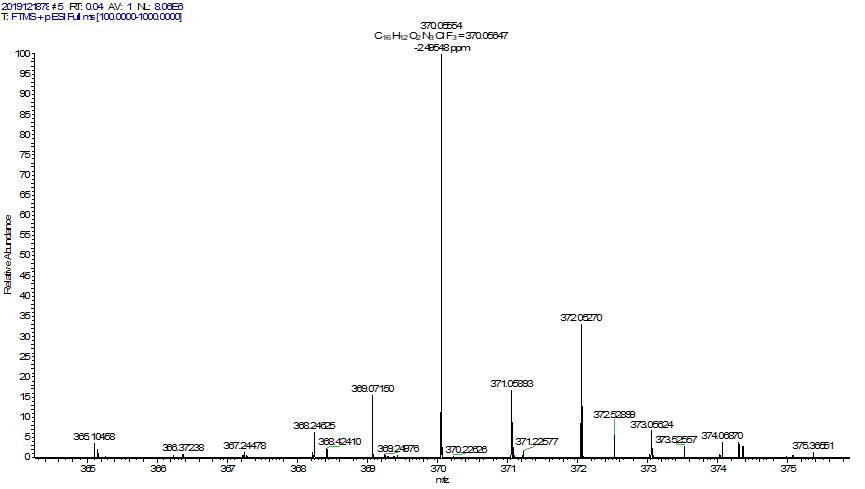


**Fig S51** HRMS of compound **5q**

**
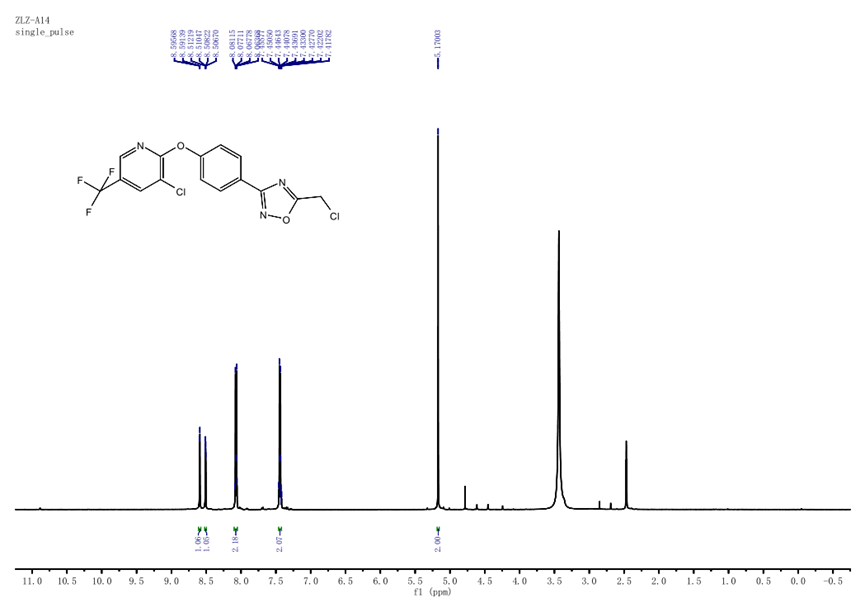
**

**Fig S52** ^1^H NMR of compound **5r**

**
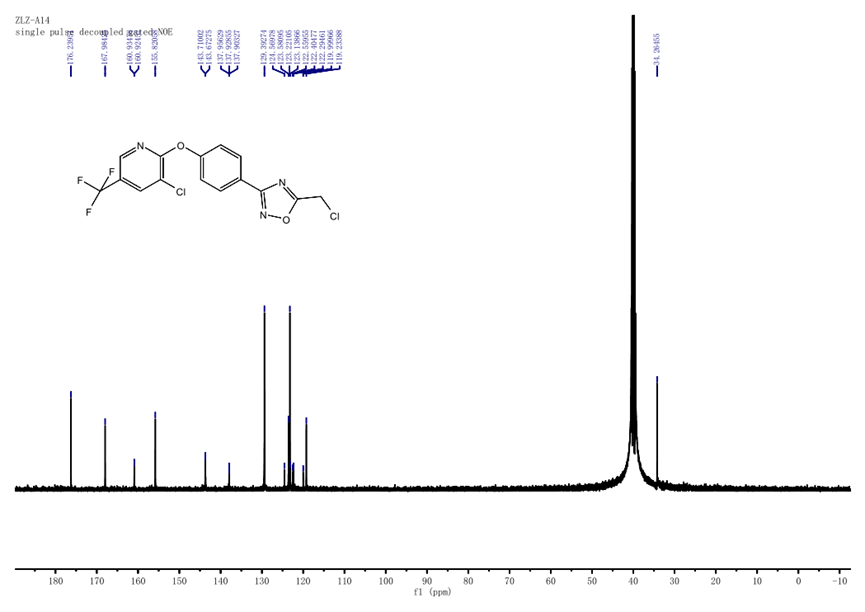
**

**Fig** S53 ^13^C NMR of compound **5r**


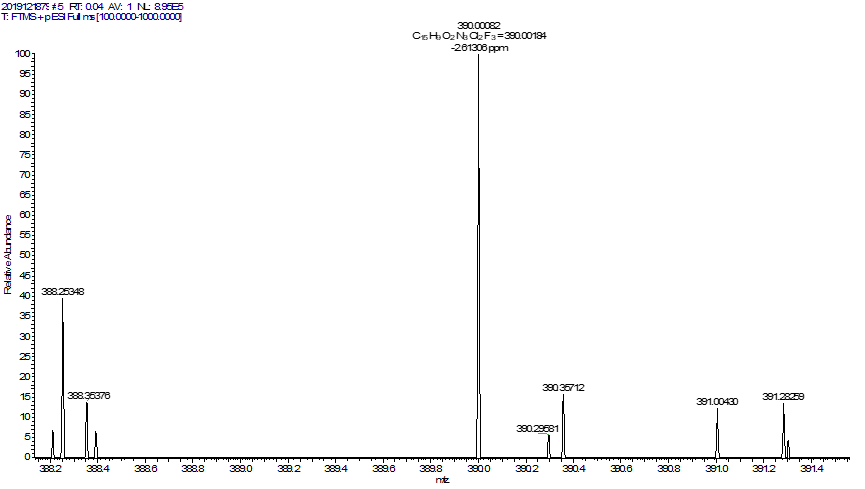


**Fig S54** HRMS of compound **5r**


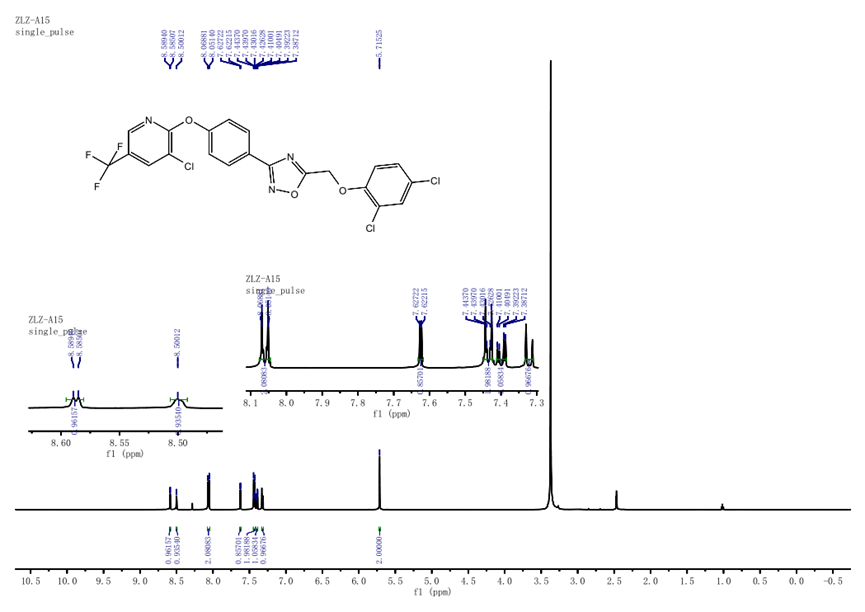


**Fig S55** ^1^H NMR of compound **5s**


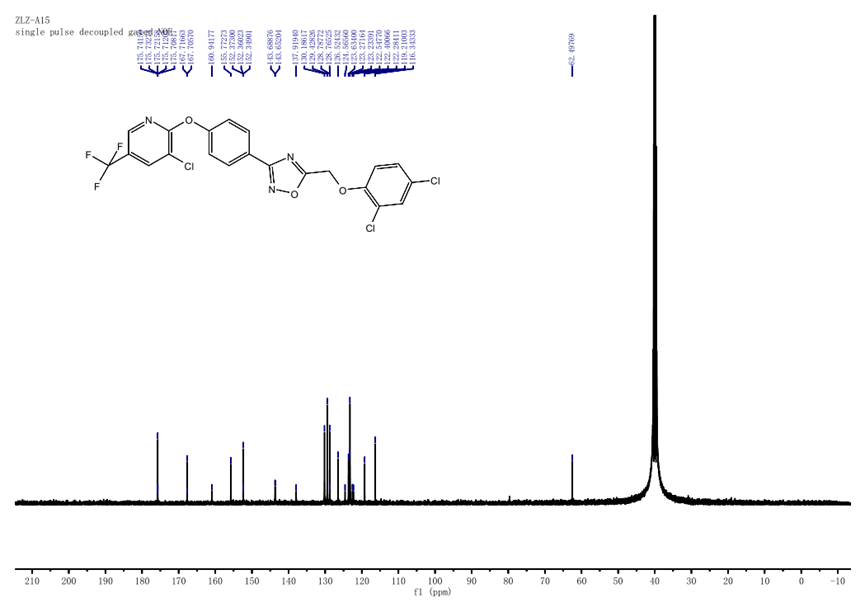


**Fig S56** ^13^C NMR of compound **5s**

**
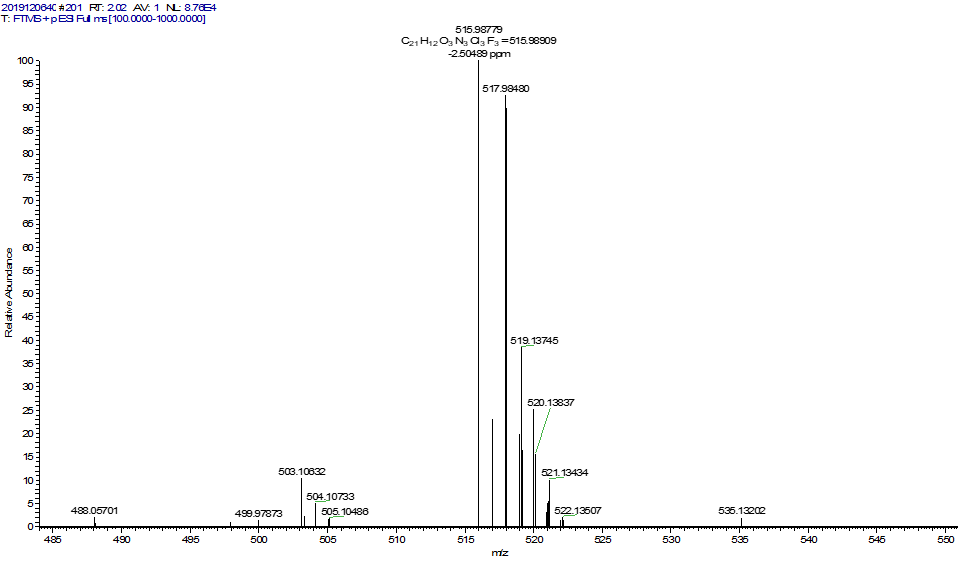
**

**Fig S57** HRMS of compound **5s**


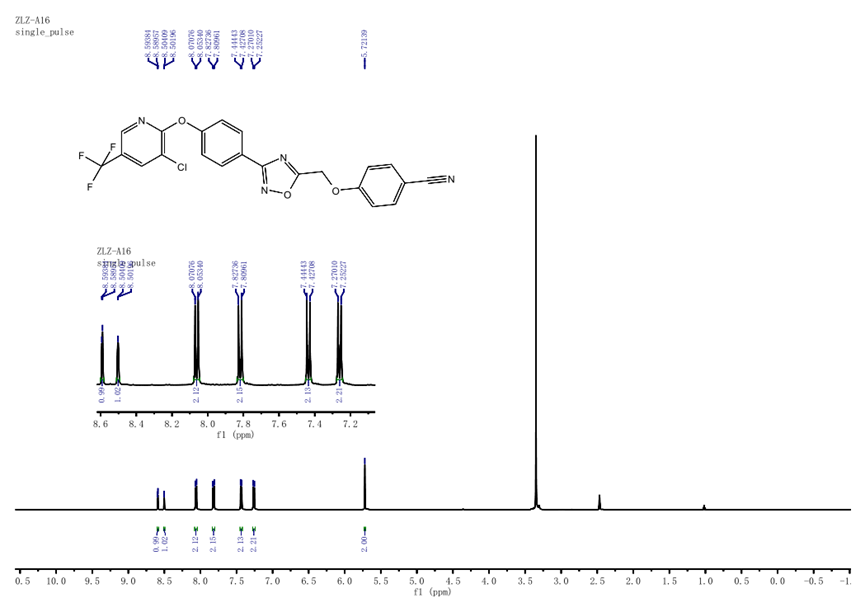


**Fig S58** ^1^H NMR of compound **5t**


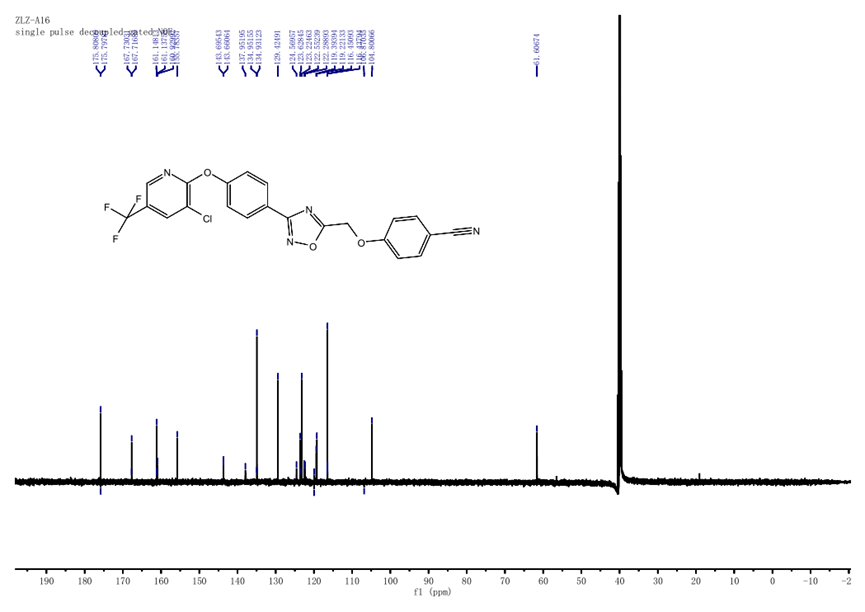


**Fig S59** ^13^C NMR of compound **5t**


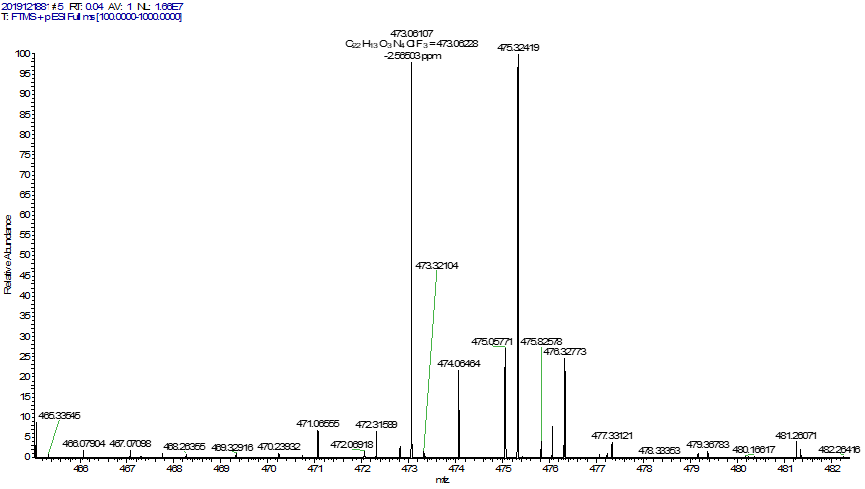


**Fig S60** HRMS of compound **5t**


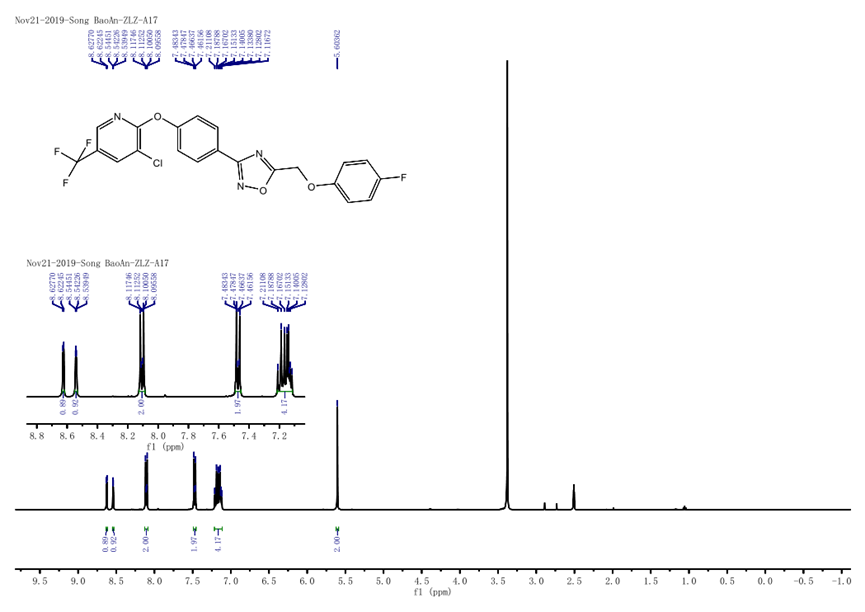


**Fig S61** ^1^H NMR of compound **5u**


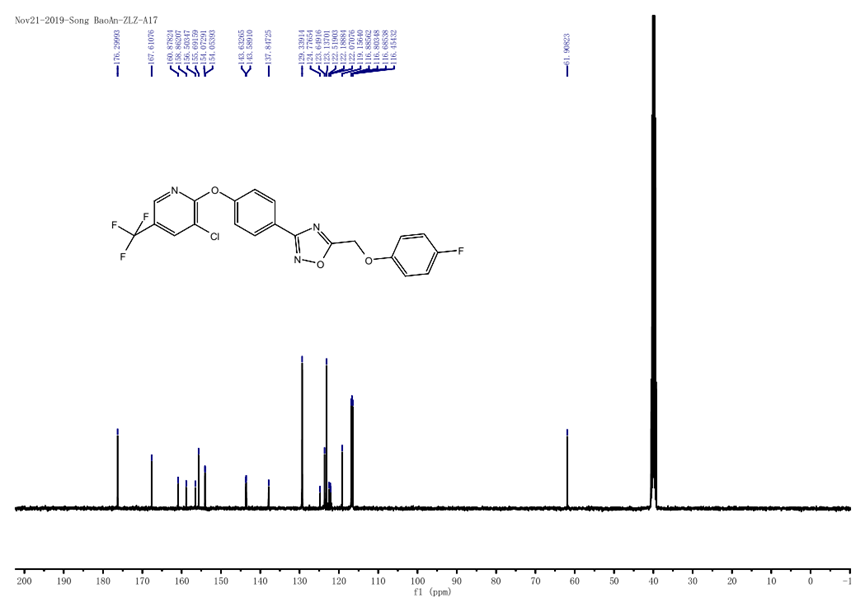


**Fig S62** ^13^C NMR of compound **5u**


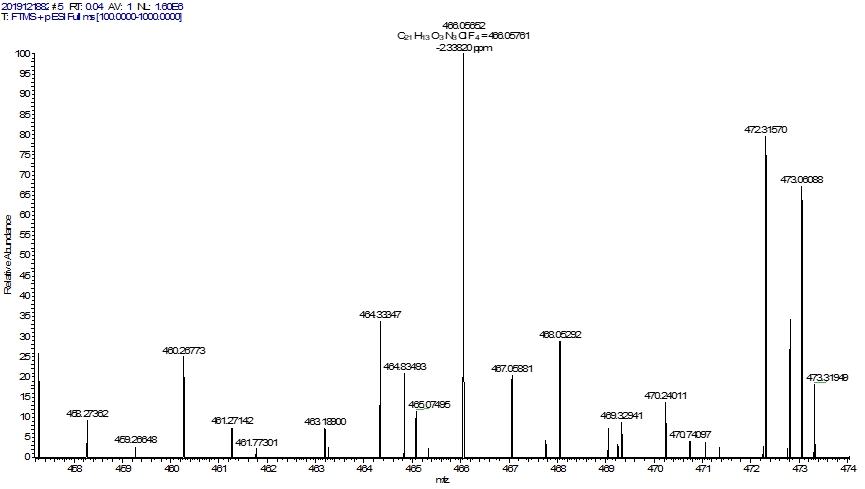


**Fig S63** HRMS of compound **5u**


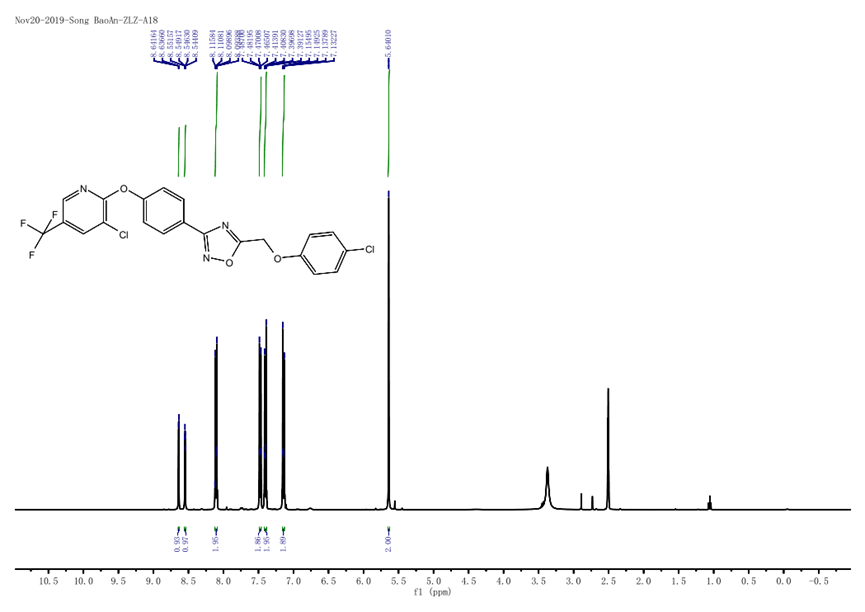


**Fig S64** ^1^H NMR of compound **5v**

**
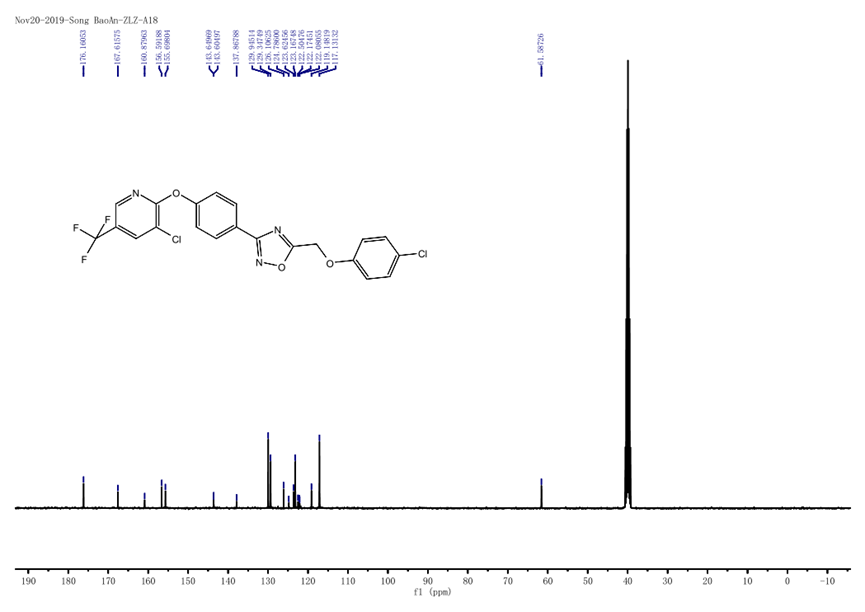
**

**Fig S65** ^13^C NMR of compound **5v**


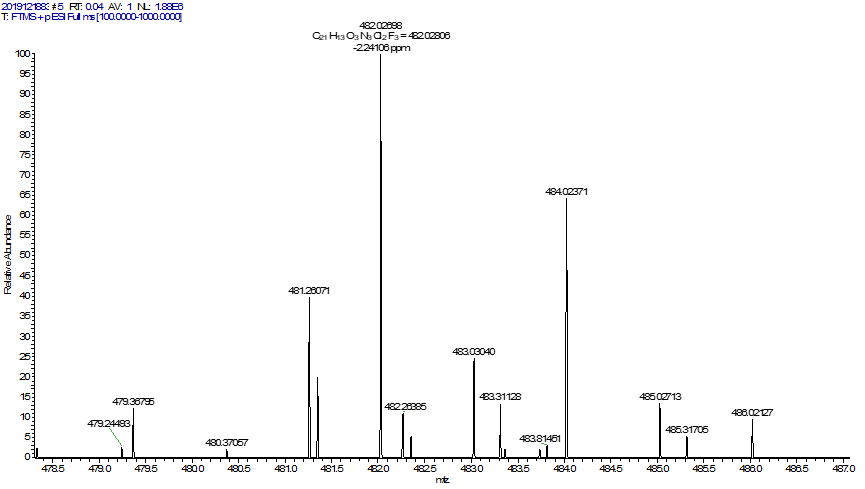


**Fig S66** HRMS of compound **5v**

**
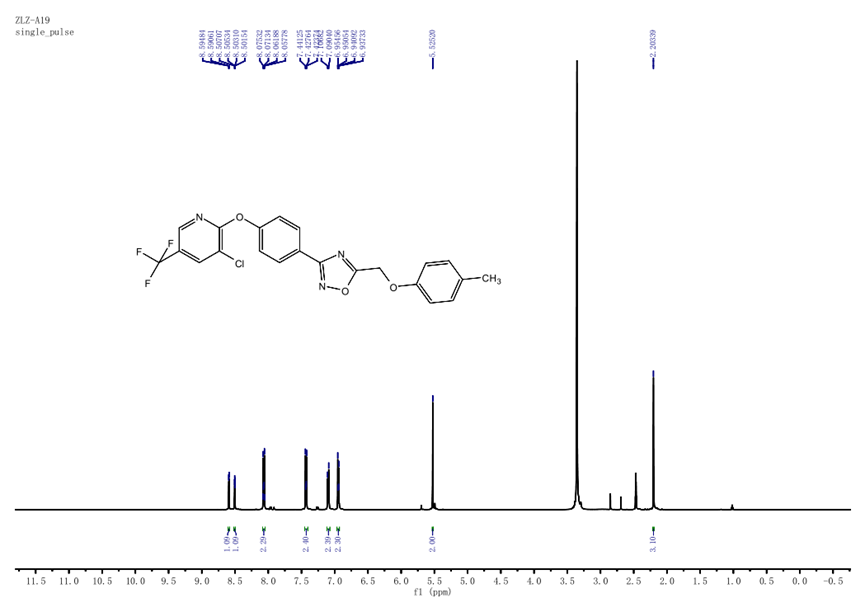
**

**Fig S67** ^1^H NMR of compound **5w**

**
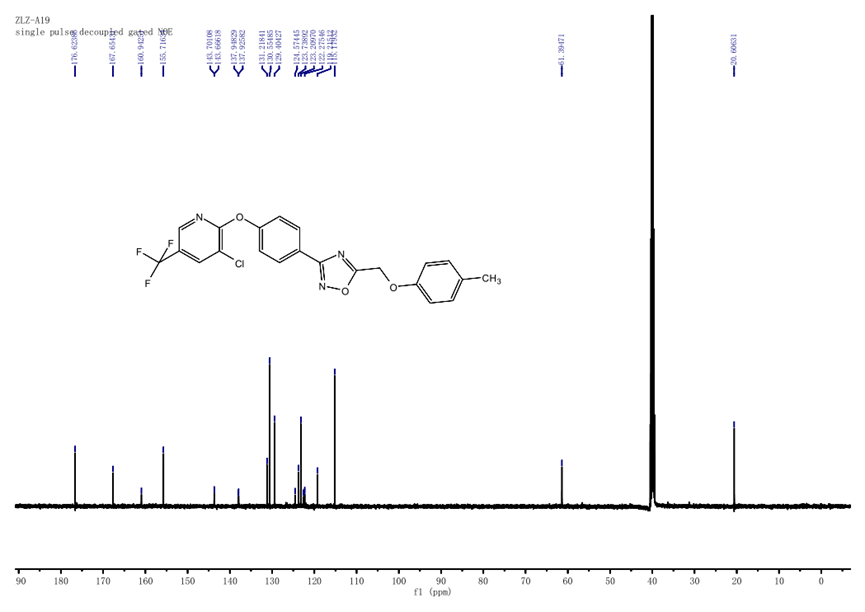
**

**Fig S68** ^13^C NMR of compound **5w**


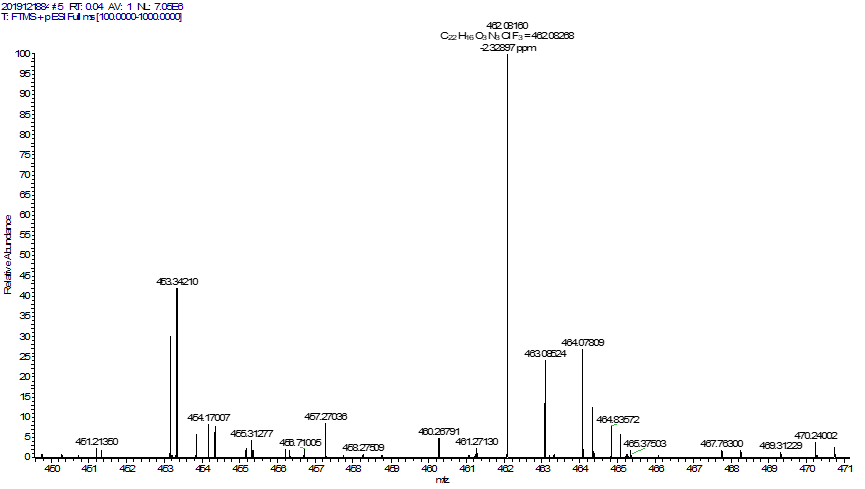


**Fig S69** HRMS of compound **5w**


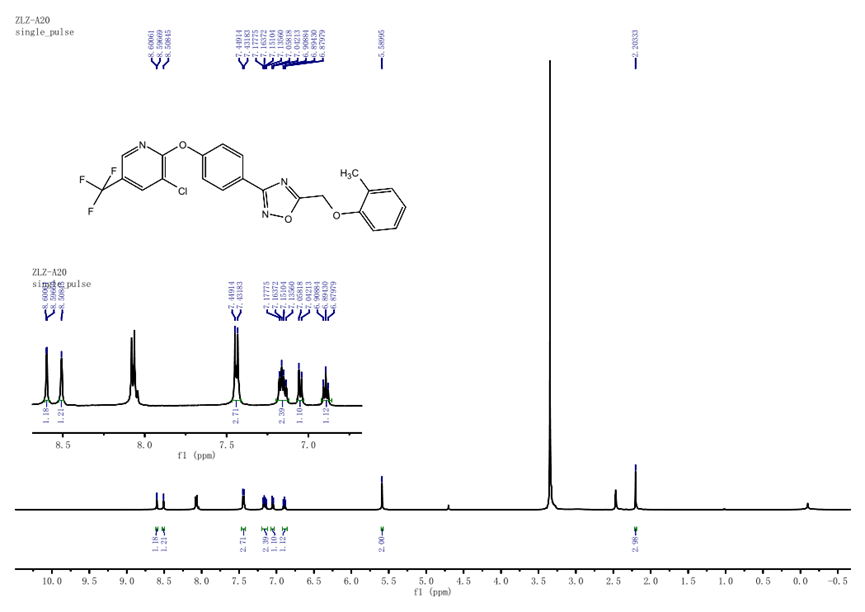


**Fig S70** ^1^H NMR of compound **5x**


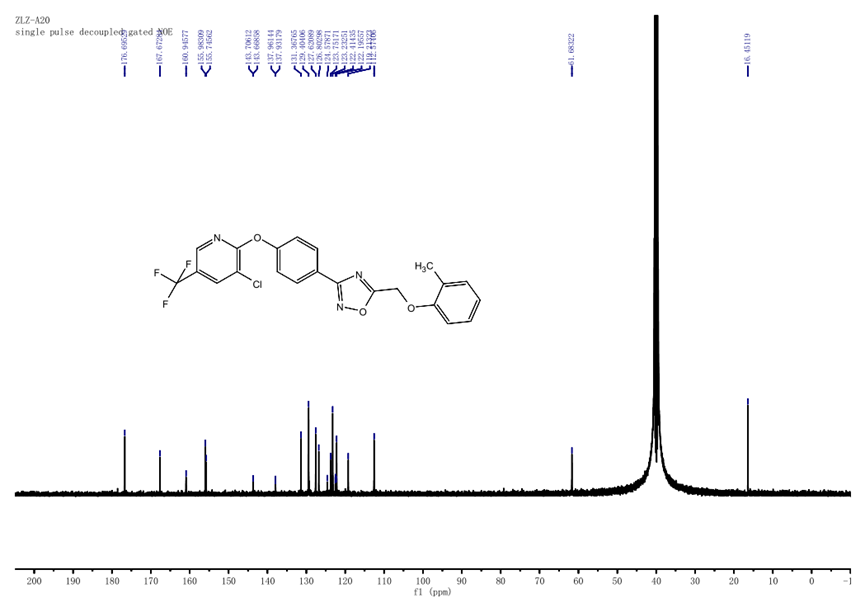


**Fig S71** ^13^C NMR of compound **5x**


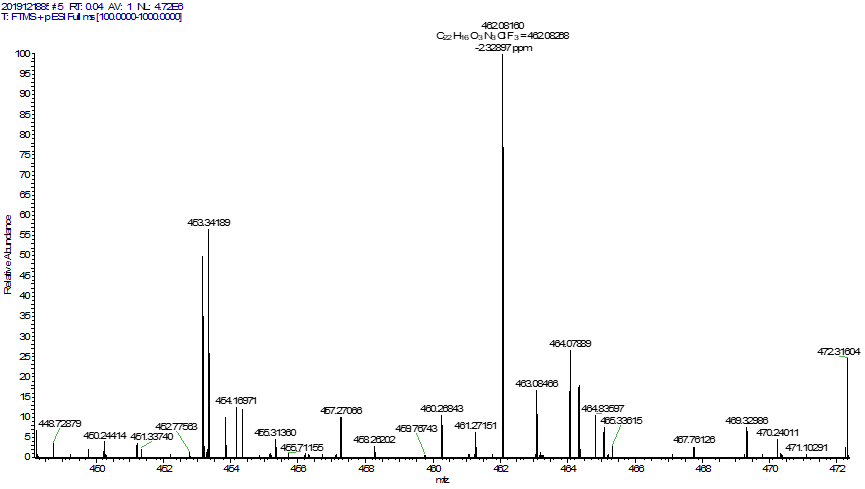


**Fig S72** HRMS of compound **5x**

**
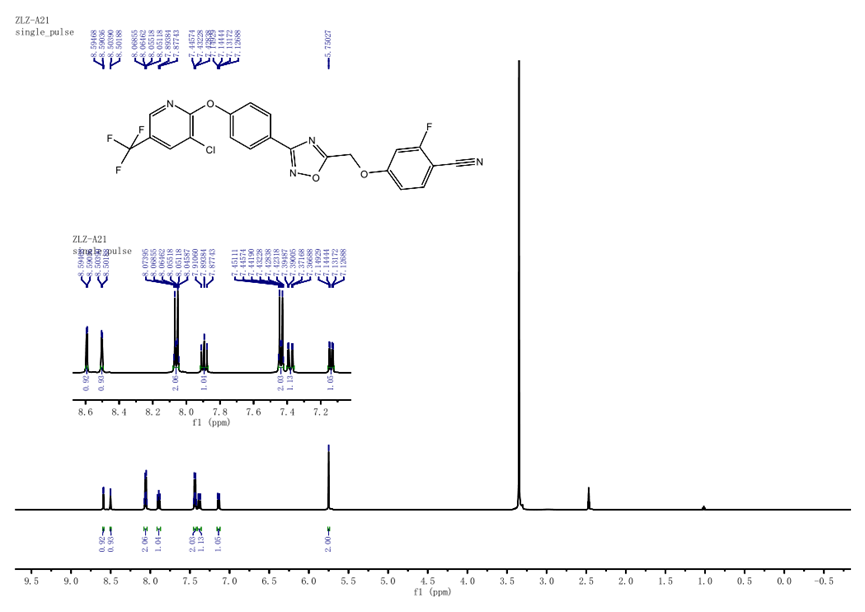
**

**Fig S73** ^1^H NMR of compound **5y**

**
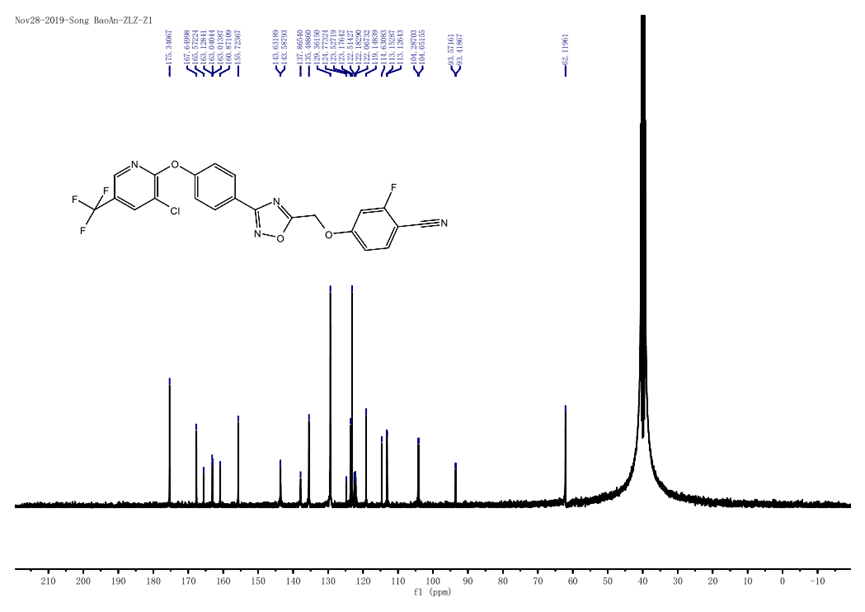
**

**Fig S74** ^13^C NMR of compound **5y**

**
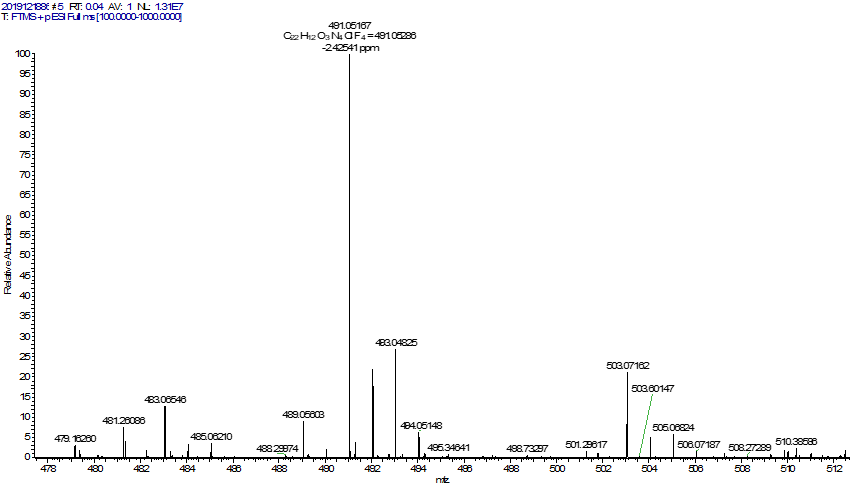
**

**Fig S75** HRMS of compound **5y**


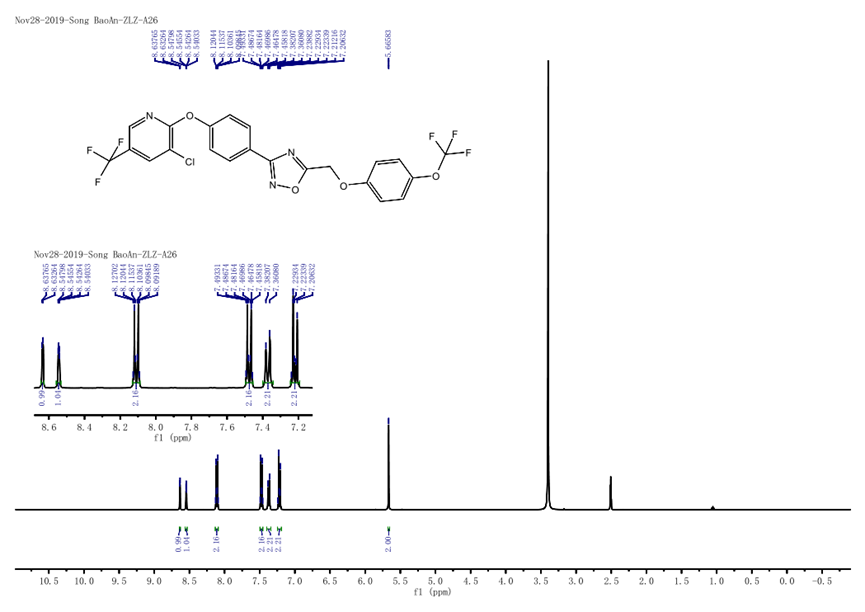


**Fig S76** ^1^H NMR of compound **5z**


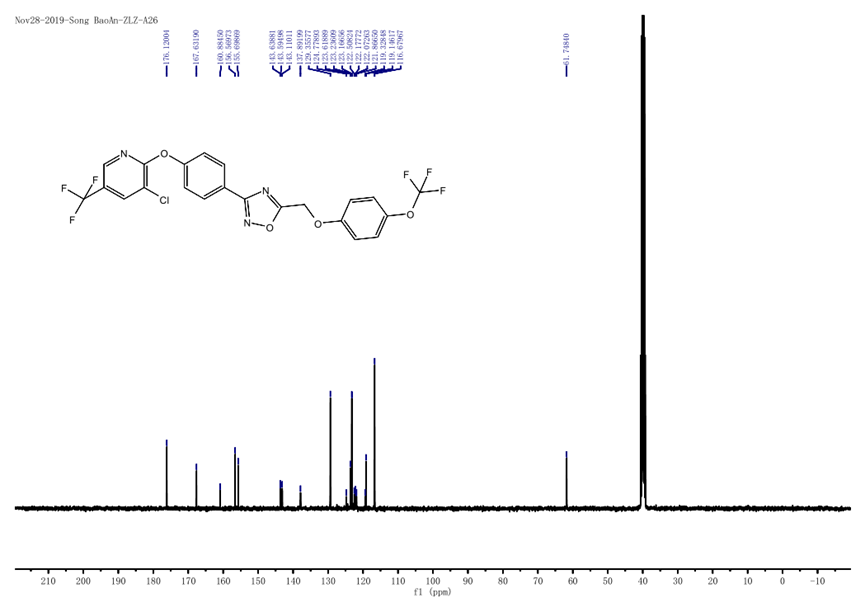


**Fig S77** ^13^C NMR of compound **5z**


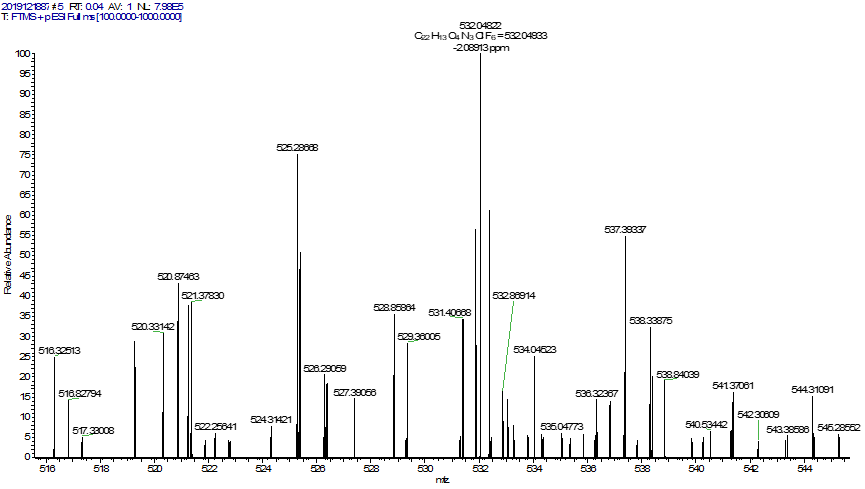


**Fig S78** HRMS of compound **5z**
